# Behavioral Balance in Tryptophan Turmoil: Regional Metabolic Rewiring in Kynurenine Aminotransferase II Knockout Mice

**DOI:** 10.3390/cells14211711

**Published:** 2025-10-31

**Authors:** Ágnes Szabó, Zsolt Galla, Eleonóra Spekker, Diána Martos, Mónika Szűcs, Annamária Fejes-Szabó, Ágnes Fehér, Keiko Takeda, Kinuyo Ozaki, Hiromi Inoue, Sayo Yamamoto, Péter Monostori, József Toldi, Etsuro Ono, László Vécsei, Masaru Tanaka

**Affiliations:** 1 HUN-REN-SZTE Neuroscience Research Group, Hungarian Research Network, University of Szeged, Danube Neuroscience Research Laboratory, H-6725 Szeged, Hungary; szabo.agnes.4@med.u-szeged.hu (Á.S.);; 2 Department of Neurology, Albert Szent-Györgyi Medical School, University of Szeged, H-6725 Szeged, Hungary; 3 Doctoral School of Clinical Medicine, University of Szeged, H-6720 Szeged, Hungary; 4 Metabolic and Newborn Screening Laboratory, Department of Pediatrics, Albert Szent-Györgyi Faculty of Medicine, University of Szeged, H-6725 Szeged, Hungary; 5 Competence Center for Drug Development and Clinical Trials, Directorate-General for Strategy and Development, University of Szeged, H-6720 Szeged, Hungary; 6 Department of Medical Physics and Informatics, Albert Szent-Györgyi Medical School, Faculty of Science and Informatics, University of Szeged, H-6720 Szeged, Hungary; 7 Competence Centre for Drug Development and Clinical Trials, Centre of Excellence for Interdisciplinary Research, Development and Innovation, University of Szeged, H-6722 Szeged, Hungary; 8 Department of Physiology, Albert Szent-Györgyi Medical School, University of Szeged, H-6720 Szeged, Hungary; 9 Department of Biomedicine, Graduate School of Medical Sciences, Kyushu University, Fukuoka 812-8582, Japan; 10 Center of Biomedical Research, Research Center for Human Disease Modeling, Graduate School of Medical Sciences, Kyushu University, Fukuoka 812-8582, Japan; 11 Department of Physiology, Anatomy and Neuroscience, Faculty of Science and Informatics, University of Szeged, H-6726 Szeged, Hungary

**Keywords:** tryptophan metabolism, kynurenine, serotonin, dopamine, kynurenine aminotransferase (KAT), oxidative stress, excitotoxicity, gut microbiota, transgenic mice, behavioral test, emotional bias, affective vulnerability

## Abstract

Background: Cognitive, emotional, and social impairments are pervasive across neuropsychiatric conditions, where alterations in the tryptophan (Trp)–kynurenine pathway and its product kynurenic acid (KYNA) from kynurenine aminotransferases (KATs) have been linked to Alzheimer’s disease, Parkinson’s disease, depression, and post-traumatic stress disorder. In novel CRISPR/Cas9-engineered KAT II knockout (*aadat^−/−^* also known as *kat2^−/−^*) mice, we observed despair-linked depression-like behavior with peripheral excitotoxicity and oxidative stress. KAT II’s role and its crosstalk with serotonin, indole-pyruvate, and tyrosine–dopamine remain unclear. It is unknown whether deficits extend to cognitive, emotional, motor, and social domains or whether brain tissues mirror peripheral stress. Objectives: Delineate domain-wide behaviors, brain oxidative/excitotoxic profiles, and pathway interactions attributable to KAT II. Results: Behavior was unchanged across strains. *kat2^−/−^* deletion remodeled Trp metabolic pathways: 3-hydroxykynurenine increased, xanthurenic acid decreased, KYNA fell in cortex and hippocampus but rose in striatum, quinaldic acid decreased in cerebellum and brainstem. These region-specific changes indicate metabolic stress across the brain and align with higher oxidative load and signs of excitotoxic pressure. Conclusions: Here, we show that KAT II deletion reshapes regional Trp metabolism and amplifies oxidative and excitotoxic imbalance. Although domain-wide behavioral measures, spanning cognition, sociability, and motor coordination, remained largely unchanged, these neurochemical alterations signify a latent emotional bias rather than overt depressive-like behavior. This work, therefore, refines prior findings by delineating KAT II–linked biochemical vulnerability as a potential substrate for stress-reactive affective dysregulation.

## 1. Introduction

Cognitive dysfunction, emotional dysregulation, motor impairment, and atypical social behavior represent core clinical features across a broad spectrum of neuropsychiatric and neurodegenerative disorders, including Alzheimer’s disease, Parkinson’s disease, schizophrenia (SCZ), and autism spectrum disorder (ASD) [1,2,3,4,5]. As the incidence of these conditions continues to rise globally, their cumulative impact on public health infrastructure, caregivers, and society becomes increasingly profound [2,3,6,7,8]. These growing challenges underscore the urgent need for elucidating the molecular and cellular mechanisms that drive these complex disorders [1,4,9,10]. Among the neurobiological systems under investigation, the metabolism of tryptophan (Trp)—an essential amino acid and a biochemical precursor to numerous neuroactive compounds—has garnered substantial attention in recent years [9,11,12,13,14].

**Figure 1:**
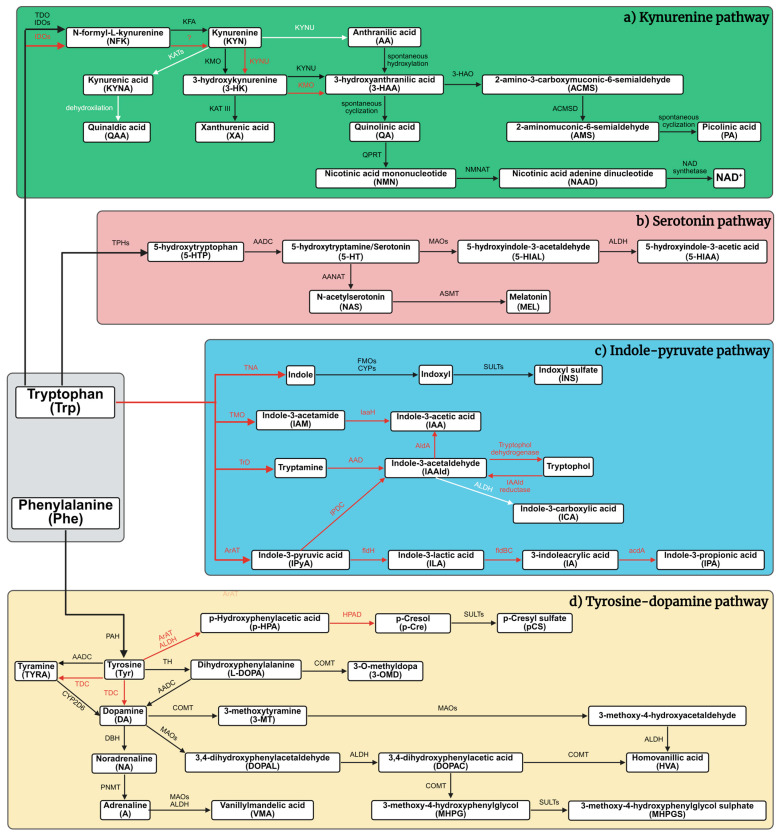
Host–microbiota co-metabolism of aromatic amino acids: tryptophan (Trp) and phenylalanine routes to neuroactive and redox-active metabolites. (**a**) Kynurenine pathway (green): tryptophan enters the kynurenine axis via TDO/IDOs to *N*-formyl-L-kynurenine → kynurenine, branching through KMO to 3-hydroxykynurenine and 3-hydroxyanthranilic acid (→ quinolinic acid) or through KATs to kynurenic acid. 2-amino-3-carboxymuconic-6-semialdehyde cyclizes/oxidizes toward quinolinic acid, which is converted by QPRT → nicotinic acid mononucleotide and onward to NAD^+^ (via NMNAT/NAD synthetase). This pathway balances neurotoxic (3-hydroxykynurenine, quinolinic acid) and neuroprotective (kynurenic acid) signals while supplying cellular NAD^+^. (**b**) Serotonin pathway (rose): tryptophan is hydroxylated by TPHs to 5-hydroxytryptophan and decarboxylated by aromatic L-amino acid decarboxylase (AADC) to serotonin. Serotonin is catabolized by MAOs/ALDH to 5-hydroxyindole-3-acetic acid, or acetylated/methylated by AANAT → N-acetylserotonin and HIOMT/ASMT → melatonin. This route links gut/brain serotonin tone with circadian signaling. (**c**) Indole-pyruvate pathway (blue; microbiota–host): bacterial TNA converts tryptophan to indole, which is oxidized to indoxyl and sulfated in the host to indoxyl sulfate. Parallel microbial transamination/reduction/oxidation steps yield indole-3-pyruvic acid → indole-3-lactic acid/indole-3-acetic acid, indole-3-acetaldehyde, 3-indoleacrylic acid, and indole-3-propionic acid. These ligands engage AhR, fortify epithelial barriers, and shape systemic immunity. Note: microbiome composition/function was not assessed here; indole readouts are interpreted as central metabolic signatures rather than direct measures of microbial activity. (**d**) Tyrosine (Tyr)–dopamine (DA) pathway (yellow): phenylalanine → tyrosine (PAH) → levodopa (TH) → dopamine (AADC) → noradrenaline (DBH) → adrenaline (PNMT), with COMT/MAOs/ALDH producing 3,4-dihydroxyphenylacetic acid, 3-methoxytyramine, homovanillic acid, vanyllilmandelic acid, and 3-methoxy-4-hydroxyphenylglycol. In parallel, microbial fermentation of phenylalanine/tyrosine generates p-Hydroxyphenylacetic acid and p-Cresol, further host-conjugated to p-Cresyl sulfate—an impactful uremic/toxic metabolite. The enzymes AADC, ArAT, MAOs, ALDH, and SULTs are involved not only in the Tyr–DA pathway but also participate in the 5-HT or indole–pyruvate metabolic pathways. Black arrows: the host routes; red arrows: the gut microbiota routes; white arrows: host and microbiota routes with the same enzyme. 3-HAA, 3-hydroxyanthranilic acid; 3-HAO, 3-hydroxyanthranilate oxidase; 3-HK, 3-hydroxykynurenine; 3-MT, 3-methoxytyramine; 3-OMD, 3-O-methyldopa; 5-HIAA, 5-hydroxyindole-3-acetic acid; 5-HIAL, 5-hydroxyindole-3-acetaldehyde; 5-HT, 5-hydroxytryptamine/serotonin; 5-HTP, 5-hydroxytryptophan; A, adrenaline; AA, anthranilic acid; AAD, amino acid decarboxylase; AADC, aromatic L-amino acid decarboxylase; AANAT, arylalkylamine N-acetyltransferase; acdA, acyl-CoA dehydrogenase; ACMS, 2-amino-3-carboxymuconic-6-semialdehyde; ACMSD, amino-*β*-carboxymuconate-semialdehyde-decarboxylase; AldA, indole-3-acetaldehyde dehydrogenase; ALDH, aldehyde dehydrogenase; AMS, 2-aminomuconic-6-semialdehyde; ArAT, aromatic amino acid aminotransferase; ASMT, acetylserotonin-O-methyltransferase; COMT, catechol-O-methyltransferase; CYP2D6, cytochrome P450 2D6; CYPs, cytochrome P450 monooxygenases; DA, dopamine; DBH, dopamine β-hydroxylase; DOPAC, 3,4-dihydroxyphenylacetic acid; DOPAL, 3,4-dihydroxyphenylacetaldehyde; fldBC, phenyllactate dehydratase; fldH, indole-3-pyruvate ferredoxin oxidoreductase; FMOs, flavin-containing monooxygenases; HPAD, 4-hydroxyphenylacetate decarboxylase; HVA, homovanillic acid; IA, 3-indoleacrylic acid; IAA, indole-3-acetic acid; IaaH, indole-3-acetamide hydrolase; IAAld, indole-3-acetaldehyde; IAM, indole-3-acetamide; ICA, indole-3-carboxylic acid; IDOs, indoleamine 2,3-dioxygenases (IDO1 and IDO2); ILA, indole-3-lactic acid; INS, indoxyl sulfate; IPA, indole-3-propionic acid; IPDC, indole-3-pyruvate decarboxylase; IPyA, indole-3-pyruvic acid; KAT III, kynurenine aminotransferase III; KATs, kynurenine aminotransferases (KAT I, II, III, and IV); KFA, kynurenine formamidase; KMO, kynurenine-3-monooxygenase; KYN, kynurenine; KYNA, kynurenic acid; KYNU, kynureninase; L-DOPA, dihydroxyphenylalanine/levodopa; MAOs, monoamine oxidases (MAO-A and MAO-B); MEL, melatonin; MHPG, 3-methoxy-4-hydroxyphenylglycol; MHPGS, 3-methoxy-4-hydroxyphenylglycol sulfate; NA, noradrenaline; NAD^+^, nicotinamide adenine dinucleotide; NAAD, nicotinic acid adenine dinucleotide; NAS, N-acetylserotonin; NFK, N-formyl-L-kynurenine; NMN, nicotinic acid mononucleotide; NMNAT, nicotinamide mononucleotide adenylyltransferase; p-Cre, p-Cresol; p-HPA, para-hydroxyphenylacetic acid; pCS, p-Cresyl sulfate; PA, picolinic acid; PAH, phenylalanine hydroxylase; Phe, phenylalanine; PNMT, phenylethanolamine N-methyltransferase; QA, quinolinic acid; QAA, quinaldic acid; QPRT, quinolinate phosphoribosyl transferase; SULTs, sulfotransferases; TDC, tyrosine decarboxylase; TDO, tryptophan-2,3-dioxygenase; TH, tyrosine hydroxylase; TMO, tryptophan-2-monooxygenase; TNA, tryptophanase; TPHs, tryptophan hydroxylases (TPH1 and TPH2); TrD, tryptophan decarboxylase; Trp, tryptophan; Tyr, tyrosine; TYRA, tyramine; VMA, vanyllilmandelic acid; XA, xanthurenic acid; ?, unknown.

Trp metabolism plays a pivotal role in modulating central nervous system (CNS) functions, particularly those related to cognitive abilities, mood regulation, and social behavior [15,16,17,18,19]. Dysregulation within these metabolic pathways has been increasingly linked to the pathophysiology of diseases marked by cognitive decline and deficits in social functioning [15,16,17,20,21]. The kynurenine (KYN) pathway is the principal route for Trp catabolism, accounting for approximately 90% of total metabolic flux [15,16,21,22,23] (Figure 1). This pathway produces a variety of bioactive metabolites with diverse effects on CNS function [15,16,18,24,25]. Among these, kynurenic acid (KYNA) stands out due to its ability to modify excitatory neurotransmission through its action on multiple receptors, including N-methyl-D-aspartate (NMDA), α7-nicotinic acetylcholine, α-amino-3-hydroxy-5-methyl-4-isoxazolepropionic acid (AMPA), and kainate receptors [24,26,27,28,29,30]. KYNA is synthesized via the irreversible transamination of KYN by kynurenine aminotransferase enzymes (KATs), with the KAT II isoform being particularly prominent in the brain [19,25,28,31,32]. In contrast, another KYN pathway metabolite, 3-hydroxykynurenine (3-HK), contributes to neurotoxicity by promoting oxidative stress through the generation of reactive oxygen species [17,20,22,33,34]. While historically KYNA and 3-HK were categorized as strictly neuroprotective and neurotoxic, respectively, emerging evidence reveals more complex, context-dependent functions that vary based on concentration, receptor expression patterns, and disease-specific factors [15,24,33,35,36].

In addition to the KYN pathway, several alternative routes for Trp metabolism significantly influence CNS homeostasis [15,37,38,39,40]. One such pathway is the 5-HT–melatonin (MEL) system [15,37,40,41,42]. 5-HT, synthesized from Trp, is a critical neurotransmitter involved in mood regulation, affective balance, and social cognition. Its downstream metabolite, MEL, regulates circadian rhythms and sleep architecture—factors integrally linked to learning, memory consolidation, and executive functioning [39,42,43,44]. Perturbations in this pathway are associated with a wide range of psychiatric disorders, including major depressive disorder (MDD), generalized anxiety disorder (GAD), and disturbances in sleep and circadian regulation [12,39,41,45,46]. Here, we quantify regional 5-HT and 5-hydroxyindoleacetic acid (5-HIAA) to estimate serotonergic turnover in vivo and relate these indices to KYN-pathway shifts.

Another prominent route is the indole–pyruvate pathway, primarily driven by the gut microbiota [47,48,49,50,51]. In this pathway, microbial enzymes convert Trp into several indole derivatives through distinct enzymatic reactions [47,48,49,51,52]. These indole metabolites are capable of crossing the intestinal barrier and influencing the CNS by modulating neuroinflammatory processes, maintaining gut epithelial integrity, and regulating blood–brain barrier (BBB) permeability [47,48,51,53,54]. This bidirectional communication along the gut–brain axis has been implicated in the pathophysiology of mood disorders, ASD, and other conditions characterized by social and emotional dysregulation [48,52,55,56,57]. We therefore profile brain-region levels of indole-3-acetic acid (IAA) and indole-3-carboxaldehyde (ICA) as sentinel markers of gut–brain indole signaling in KAT II knockout mice. ICA, IAA, and indoxyl sulfate (INS) are established readouts of microbiota-derived indole flux, but microbiome profiling was not performed here; thus, brain-region values represent neurochemical correlates rather than direct measures of microbial composition or function.

Additionally, Trp metabolism exerts regulatory effects on dopaminergic neurotransmission via its influence on the tyrosine (Tyr)–dopamine (DA) pathway [37,58,59,60,61]. Specifically, Trp availability impacts the synthesis of tetrahydrobiopterin (BH4), a critical cofactor required by tyrosine hydroxylase—the rate-limiting enzyme in DA production [37,58,59,62,63]. Given DA’s fundamental role in mediating reward processing, attentional control, and social engagement, this intersection further emphasizes the extensive reach of Trp metabolism in orchestrating complex behavioral and cognitive outcomes [39,59,64,65,66]. To capture this crosstalk, we quantify Tyr, levodopa (L-DOPA), DA, and downstream metabolites alongside the pterin pool (BH4, dihydrobiopterin [BH2], and biopterin [BIO]).

In earlier research, we examined the interrelationships between affective disorders—such as MDD, GAD, and post-traumatic stress disorder—and systemic alterations in Trp and its downstream metabolites in the *kat2^−/−^* mice model [67]. Although traditionally conceptualized as mood disorders, these conditions also encompass profound cognitive impairments, including deficits in memory, sustained attention, and executive functioning [68,69,70,71,72]. Such impairments frequently manifest early in the course of illness and may persist independently of affective symptoms [68,70,72,73,74]. Notably, MDD has emerged as a significant risk factor for the subsequent development of neurodegenerative conditions like AD [68,70,75,76,77]. In parallel, individuals suffering from MDD and GAD often exhibit marked social dysfunction, including social withdrawal, blunted affect, and reduced empathic capacity [68,69,71,72,78]. These features closely parallel behavioral phenotypes observed in ASD and SCZ, further complicating differential diagnosis and therapeutic decision-making [68,71,78,79,80,81]. A mechanistic understanding of the molecular pathways that underlie these shared features is therefore of paramount importance [68,75,81,82,83].

To probe the contributions of Trp metabolic dysregulation to these behavioral phenotypes, we utilized a genetically engineered mouse model deficient in KAT II (*kat2^−/−^*) [67]. This knockout model disrupts the biosynthetic pathway for KYNA, allowing for detailed investigation of downstream metabolic consequences. Targeted metabolomic profiling of urine and plasma revealed a pronounced decrease in KYNA levels, accompanied by elevated concentrations of 3-HK. These findings reinforce the essential role of KAT II in modulating the balance between neuroprotective and neurotoxic metabolites within the KYN pathway. Building directly on this peripheral signature, the current study extends metabolomics to five brain regions (striatum [STR], cortex [CTX], hippocampus [HIPP], cerebellum [CER], brainstem [STEM]) to resolve central, region-specific consequences of KAT II deletion.

Despite inherent limitations in modeling human psychiatric and neurodegenerative diseases in rodents—especially with regard to complex cognitive processes and nuanced social behaviors—this genetic model affords a valuable platform for dissecting the neurochemical substrates of behavior [84,85,86,87,88]. In the present study, we aimed to systematically evaluate cognitive function and social behavior in *kat2^−/−^* mutant mice, with a particular focus on mapping these behavioral parameters to region-specific changes in the concentrations of Trp and its metabolites within the brain. To avoid overreach, links to depression and post-traumatic stress disorder are framed at the pathway level rather than the disorder level, recognizing that this model does not reproduce full clinical syndromes. To enhance cross-domain alignment, behavioral endpoints are mapped to region-specific metabolic indices using shared labels and synchronized panel order across figures and tables, with NORT and 3CT panels cross-referenced to cortical and hippocampal KYNA, 3 HK, and XA, and Rotarod aligned to striatal metrics. This integrative approach offers a robust framework for uncovering potential neurochemical signatures that underlie cognitive and social impairments. Accordingly, our prespecified objectives were to (i) map region-resolved Trp metabolism across the KYN, 5-HT, and indole axes; (ii) quantify the Tyr to L-DOPA to DA cascade and its enzymatic and cofactor milieu (BH4, BH2, BIO); (iii) infer pathway activities using product-substrate ratios (for example, KMO and KAT fluxes, monoamine oxidase [MAO] and aldehyde dehydrogenase [ALDH] turnover) and derive oxidative-stress and excitotoxicity indices; (iv) link these neurochemical states to a broadened behavioral battery encompassing cognition (novel object recognition [NORT], object-based attention [OBAT], Y-maze test), motor coordination (rotarod test), emotion (marble burying test [MBT]), and sociability (three-chamber test [3CT]); and (v) test concordance between brain and peripheral metabolic signatures. By coupling this expanded behavioral panel with multi-region neurochemical profiling, we aim to delineate how KAT II loss reshapes a KYN-tilted, cofactor-constrained, and indole-modulated milieu, and to determine whether such biochemical disequilibria necessarily generalize to global cognitive or social dysfunction, thereby informing pathway-targeted therapeutic strategies across neuropsychiatric spectra [89]. We therefore consider whether regionally divergent KYN remodeling could preserve baseline performance through circuit-level buffering while predisposing selected behavioral domains to failure under cognitive load or stress.

In our previous investigation [67], *kat2^−/−^* mice exhibited despair-like responses under stress-inducing paradigms such as the forced swim test, suggesting enhanced affective vulnerability. The present study, however, was specifically designed to determine whether these affective alterations persist under non-stressful baseline conditions and extend to cognitive, social, and motor domains. The absence of significant behavioral divergence observed here indicates that KAT II deficiency alone does not elicit broad behavioral dysfunction but may instead confer a latent predisposition that becomes evident only under environmental or metabolic stress. This distinction refines our earlier interpretation by differentiating stress-contingent affective reactivity from baseline behavioral stability.

## 2. Materials and Methods

This study used a standardized behavioral battery (NORT, OBAT, Y-maze, marble burying, three-chamber, rotarod) with targeted ultra-high-performance liquid chromatography with tandem mass spectrometry (UHPLC–MS/MS) metabolomics across five brain regions. Reporting followed ARRIVE 2.0 guidelines; genotypes were confirmed by a TaqMan allelic discrimination assay on HotSHOT-extracted DNA. Behavioral readouts were acquired with automated video tracking (EthoVision XT14, Noldus Information Technology BV, Wageningen, The Netherlands), and metabolite quantification employed isotope-labeled internal standards and established multiplex panels. Enzyme-activity proxies and oxidative/excitotoxic indices were computed from product–substrate ratios. Statistical analysis prespecified normality testing, variance checks, outlier detection, effect sizes (Hedges g with bootstrap CIs), and post hoc power. Methodological choices align with validated OBAT/NORT protocols, EthoVision reliability, and best practices for UHPLC–MS/MS quantitation and animal reporting.

### 2.1. Ethical Approval

The Department of Nature Conservation of the Ministry of Agriculture authorized the use of genetically modified organisms in a level 2 biosafety closed system (permit number: TMF/43-20/2015). The import of genetically modified animals was approved by the Department of Biodiversity and Gene Conservation of the Ministry of Agriculture (permit number: BGMF/37-5/2020). The investigations were conducted in accordance with the Ethical Codex for Animal Experiments and were approved by the Ethics Committee of the Faculty of Medicine at the University of Szeged, as well as by the National Food Chain Safety Office, under permission number XI./84/2025. and XI./1008/2025, in accordance with Government Decree 40/2013 (II.14.), and the European Communities Council Directive 2010/63/EU.

### 2.2. Animals

The C57BL/6N wild-type (WT) strain was originally sourced from Charles River Germany. The *kat2^−/−^* strain was provided by our collaboration partners at Kyushu University (Fukuoka, Japan). A comprehensive description of the generation of the genetically modified strain can be found in our previously published article [67]. The animals were housed in groups of 4–5 per cage in polycarbonate enclosures (530 cm^2^ floor area) under specific pathogen-free conditions at the Animal Facility of the Department of Neurology, University of Szeged. Environmental parameters were stringently controlled, with ambient temperature maintained at 24 ± 1 °C and 45–55% relative humidity under a 12:12 h light–dark cycle. Throughout the duration of the investigation, mice had unrestricted access to standard rodent food and water. Environmental enrichment was provided using paper rolls, gnawing wood, and nesting cotton. In total, 46 WT and 49 *kat2^−/−^* mice were included in the behavioral assessments, while an additional cohort of 10 WT and 10 *kat2^−/−^* mice was used for metabolomic analyses. The general condition of the animals was monitored weekly until the start of the experiments, and daily during the experimental period, using a standardized scoring system. This system was applied to assess body weight, general appearance, respiration, mobility, and basic reflexes. If an animal were to reach the predetermined critical score threshold, it would be humanely withdrawn from the study.

### 2.3. Genotyping with Taqman Allelic Discrimination Assay

All animals were genotyped prior to enrollment. Tail biopsies were collected under 2% isoflurane anesthesia with topical lidocaine and processed by an alkaline lysis protocol adapted from HotSHOT. The extraction yielded DNA suitable for downstream analysis. Concentration and purity were verified by spectrophotometry, and extracts were stored at −20 °C until use [89].

Genotypes were determined with a TaqMan allelic discrimination assay on a CFX Opus 96 real-time PCR system (Bio-Rad Laboratories, Hercules, CA, USA). Reactions were run in singleplex with allele-specific primers and dual-labeled probes. Each plate contained non-template controls together with verified WT and *kat2^−/−^* controls. Allele calls were assigned from endpoint fluorescence scatter plots and were cross-checked by amplification curves. Ambiguous calls were repeated from the DNA stock.

To improve readability, only the assay overview is retained in the main text. Complete procedural details, including primer and probe sequences, reagent compositions, thermal cycling parameters, plate layout, cluster calling criteria, and representative allelic discrimination plots, are provided in the Supplementary Materials.

### 2.4. Behavioral Tests

Cognitive, emotional, motor, and social domains were assayed with NORT for recognition memory, OBAT for attention, Y-maze spontaneous alternation for working memory, MBT, the accelerating rotarod for motor coordination, and 3CT for sociability. The behavioral experiments were performed on 8-week-old male mice of the C57BL/6N and *kat2^−/−^* strains, with *n* = 10–13 animals included per group. Sample sizes were determined using a power analysis performed with the GPower 3.1 statistical software. A *t*-test (difference between two independent means, two groups, two-tailed) was applied with the following parameters: significance level (α) = 0.05, power (1–β) = 0.8, effect size d = 1.33, and allocation ratio N2/N1 = 1. Based on these calculations, the required sample size for the behavioral tests was *n* = 10 per group. After completing the tests, a post hoc analysis was performed to verify whether the sample size was adequate for detecting large effect sizes. The following results were obtained: normality parameter = 2.973, critical t = 2.100, Df = 18, yielding an achieved power of 0.802. Based on these calculations, the resulting statistical power was approximately 0.80, indicating that under the given assumptions, the sample size was sufficient to detect large effect sizes. The animals were habituated to handling by the experimenters for one week prior to testing. all tests were conducted between 8:00 a.m. and 12:00 p.m. Prior to testing, animals were transferred to the experimental laboratory one hour in advance. NORT, OBAT, Y-maze, MBT, and 3CT were recorded using a video tracking system (Basler ace Classic acA1300-60 gm, Basler AG, Ahrensburg, Germany) in combination with behavioral analysis software (EthoVision XT14, Noldus Information Technology BV, Wageningen, The Netherlands). For the rotarod test, we used the TSE RotaRod V4.2.6 system (TSE Laboratory, Ormskirk, UK).

#### 2.4.1. Novel Object Recognition Test (NORT)

For the NORT, we used *n* = 12 animals per group (total of 24 animals). The behavioral assessments were conducted in a 60 × 60 × 60 cm open-field arena. Three distinct objects—different in color and shape but matched in size and scale relative to the animals—were utilized. The test was carried out across three consecutive days [90,91,92,93,94]. On the habituation day, each animal was placed in the empty arena for a duration of 10 min on the second day (training session), animals were allowed to explore two of the three objects for 10 min, Animals that failed to exhibit any interaction with the object designated as the familiar object during the training phase were excluded from further analysis in the experiment. On the third day (test session), one of the familiar objects from the training phase was substituted with the previously unencountered third object. This unfamiliar item functioned as the novel object, whereas the remaining object served as the familiar object. During NORT, we measured the following parameters: (1) time spent with the training object in the training phase, (2) time spent with the familiar object in the training phase, (3) time spent with the familiar object in the testing phase, (4) time spent with the novel object in the testing phase.

During both NORT and OBAT, the duration of investigation directed toward the novel and familiar objects was systematically recorded. object recognition and novelty preference, two normalized metrics were employed: the discrimination index (DI) and the preference index (PI). The DI quantifies the relative preference for the novel object compared to the familiar one, while the PI expresses the proportion of total exploration time that the animal allocated to the novel object. Both indices account for individual variability in total investigation time and were computed using the following formulas (Equations (1) and (2)).

(1) $$discrimination\;index\left(DI\right)=\frac{Tnovel-Tfamiliar}{Tnovel+Tfamiliar}$$

(2) $$preference\;index\left(PI\right)=\frac{Tnovel}{Tnovel+Tfamiliar}\times 100$$

#### 2.4.2. Object-Based Attention Test (OBAT)

The object-based attention test (OBAT), originally developed by Wulaer and colleagues, represents a validated behavioral paradigm for evaluating attentional performance in rodents [95,96,97,98,99]. This method is similar to the NORT; the OBAT leverages the rodent’s intrinsic exploratory drive and preference for novelty. A total of 24 animals were used, with 12 assigned to each group (*n* = 12). The experimental setup comprises a two-compartment arena with dimensions of 40 × 40 × 40 cm (larger compartment) and 20 × 40 × 40 cm (smaller compartment) and utilizes six distinct objects. These objects differ in color and shape but are comparable in size. The procedure consists of two sequential phases: a training phase and a testing phase. During the training phase, the animal is introduced into the larger compartment containing five distinct objects and is allowed a 3 min exploration period. Animals that did not engage in any interaction with the object assigned as the familiar object during the training phase were excluded from subsequent experimental analysis. Subsequently, one of these previously encountered objects, along with a sixth, novel object, is placed in the smaller compartment for the 3 min test phase. The novel item serves as the novel object, while the reintroduced item functions as the familiar object. Animals that did not engage with the object, later serving as the familiar stimulus during the training phase, were systematically excluded from subsequent experimental evaluation.

The following parameters were assessed during the NORT: (1) duration of interaction with the training objects during the training phase, (2) duration of interaction with the familiar object during the training phase, (3) time spent exploring the familiar object during the testing phase, and (4) time spent interacting with the novel object during the testing phase.

#### 2.4.3. Y-Maze Test

Rodents exhibiting intact working memory capacity, and thereby preserved prefrontal cortical function, are capable of recalling which arms have been recently explored and display a preferential inclination to enter the arm that has not been visited in the most recent sequence [100,101,102,103]. A total of 24 animals were used, with 12 assigned to each group (*n* = 12). At the onset of the trial, the animal is positioned at the distal end of the longest arm of the Y-maze, oriented toward the central zone. Thereafter, it is granted an eight-minute period to freely explore the maze. The spontaneous alternation rate was determined according to the following formula (Equation (3)).

(3) $$spontaneous\;alternation\left(\%\right)=\frac{number\;of\;spontaneous\;alternations}{total\;number\;of\;arm\;entries-2}\times 100$$

During the Y-maze test, we measured spontaneous alternation behavior as well as the total number of entries into all three arms.

#### 2.4.4. Marble Burying Test (MBT)

The marble burying test (MBT) was used to evaluate repetitive and compulsive-like behaviors. Although its interpretation remains debated, several studies suggest that increased marble burying may be associated with enhanced behavioral rigidity and social withdrawal, particularly in animal models displaying impaired sociability [104,105,106,107,108].

The MBT was conducted with 23 animals in total, including 10 WT and 13 *kat2^−/−^* mice (*n* = 10–13 per group). The animals were individually placed in a transparent plastic arena measuring 40 × 24 × 18 cm. The base of the apparatus was filled with a 5 cm deep layer of fresh bedding material. To allow adequate ventilation while preventing escape. The enclosure was covered with a transparent plastic lid (40 × 24 cm, 1 cm thick) featuring six circular perforations, each 1 cm in diameter. At one end of the arena, sixteen glass marbles (each with dimensions of 1 × 1 × 1 cm^3^) were positioned on the bedding in a regular square grid formation. The outermost marbles were placed 3.5 cm from the arena walls, with 5 cm spacing between adjacent marbles. Each mouse was allowed to explore the arena freely for a period of 30 min. The marbles were categorized based on their status: intact, displaced, partially buried (0–75%), or fully buried (75–100%). These measurements were subsequently analyzed and compared across experimental groups.

#### 2.4.5. Three Chamber Test (3CT)

Sociability is operationally defined as the propensity of the test mouse to spend a greater proportion of time in the compartment containing a novel conspecific, as opposed to the compartment housing a novel inanimate object. A supplementary and confirmatory metric involves the quantification of time spent engaging in olfactory investigation of the novel conspecific relative to the novel object, thereby providing an index of direct social interaction. Additionally, the number of transitions between compartments [109,110,111,112,113].

A total of 24 animals were used, with 12 assigned to each group (*n* = 12). A rectangular three-chambered apparatus was employed for the 3CT. Each compartment measured 20 × 40.5 × 22 cm. The chambers were divided by opaque gray plastic walls, each containing manually operated doors (7.5 × 5 cm) to allow controlled access between compartments. Cylindrical wire-mesh enclosures (15 cm in height, 7 cm in diameter) were positioned in both lateral chambers. The mesh structure, composed of bars spaced 1 cm apart, permitted adequate airflow between the interior and exterior of the cylinder while simultaneously preventing direct physical contact between the test subject and the stimulus animal or object placed within. This configuration allowed for the assessment of social preference and investigatory behavior while minimizing confounding factors related to tactile interaction [109,114,115,116,117]. The test protocol consisted of three distinct phases. During the habituation phase, the subject mouse was confined to the center chamber of the apparatus for a period of 10 min with all doors closed, allowing acclimatization to the environment. In the sociability phase, the doors to the lateral chambers were opened, and the test mouse, starting from the center chamber, was allowed to freely explore all three compartments for 10 min. One lateral chamber contained an empty wire-mesh enclosure, while the other housed a wire cage enclosing a novel conspecific that was matched to the test animal in sex, age, and body weight. The social novelty preference phase followed a similar structure: the subject animal started from the center chamber and was given 10 min to explore the entire apparatus. In this phase, the previously encountered conspecific from the sociability phase served as the familiar animal, while a non-familiar, sex-, age-, and weight-matched conspecific was introduced into the formerly empty cage, serving as the novel animal.

During the sociability phase, we quantified (1) time spent in the social chamber, (2) time spent in the non-social chamber, (3) time spent in the center chamber, (4) time spent sniffing the social cage, (5) time spent sniffing the non-social cage, (6) number of entries to the social chamber, (7) number of entries to the non-social chamber, (8) number of entries to both chambers. In the social novelty preference phase, we measured the (1) time spent in the novel chamber, (2) time spent in the familiar chamber, (3) time spent in the center chamber, (4) time spent sniffing the novel animal’s cage, (5) time spent sniffing the familiar animal’s cage, (6) number of entries to the novel chamber, (7) number of entries to the familiar animal’s chamber, (8) number of entries to both chambers.

#### 2.4.6. Rotarod Test

The apparatus comprised a rotating rod equipped with an automated fall-detection system at the base, which interfaced with the TSE RotaRod V4.2.6 system (TSE Laboratory, Berlin, Germany) to automatically terminate the timer upon the animal’s fall [118,119,120,121,122]. We used *n* = 12 animals per group (a total of 24 animals). Animals underwent a two-day habituation and training protocol. On Day 1, each mouse was placed individually on the rotating rod, which was maintained at a constant speed of five revolutions per minute (rpm) for a duration of three minutes. Should the animal have fallen before the allotted time elapses, it was promptly repositioned on the rod. Following the initial session, the animal was returned to its home cage, and the procedure was repeated twice more at 30 min intervals. On Day 2, the training procedure was repeated, with the rotation speed increased to a constant 10 rpm, thereby introducing a higher motoric challenge and reinforcing task familiarity. The testing phase was conducted on Day 3. During this phase, animals were placed on the rod, which now accelerated linearly from 5 to 40 rpm over a 3 min period. Each animal underwent three test trials, with a 30 min inter-trial interval. Unlike during training, animals were not returned to the rod after falling. The latency to fall—defined as the time the animal remained on the rod before falling—was recorded automatically via the tracking software. The average latency across the three test trials served as the composite performance score for each subject, reflecting overall motor coordination and skill retention under increasing demands.

### 2.5. Ultra-High-Performance Liquid Chromatography with Tandem Mass Spectrometry (UHPLC-MS/MS)

#### 2.5.1. Brain Samples

A total of *n* = 10 animals per group were included in the metabolomic measurements. Sample sizes were estimated through power analysis using GPower 3.1 statistical software. Calculations were based on a two-tailed *t*-test comparing two independent means, with the following parameters: significance level (α) = 0.05, power (1–β) = 0.8, effect size (d) = 1.33, and an allocation ratio (N2/N1) of 1. According to these estimates, a sample size of *n* = 10 animals per group was required for the measurements. Following the completion of the measurements and statistics, a post hoc power analysis was conducted to assess whether the sample size was sufficient to detect large effect sizes. The analysis yielded the following parameters: normality = 2.973, critical t = 2.100, and degrees of freedom (Df) = 18, resulting in an achieved power of 0.802. These findings indicate that, under the given assumptions, the statistical power was approximately 0.80, confirming that the sample size was adequate for detecting large effect sizes.

For tissue collection, mice were anesthetized with 2% isoflurane and then perfused transcardially with artificial cerebrospinal fluid. The brains were subsequently dissected into five distinct regions—striatum, cortex, hippocampus, cerebellum, and brainstem. All tissues were collected on ice and stored at −80 °C until further analysis. Tissue sampling was performed between 8 am and 12 pm local time to limit circadian variability in Trp pathway measures.

We used a randomized, blinded, region-resolved design that integrates targeted liquid chromatography with tandem mass spectrometry (LC-MS/MS) metabolomics with a standardized behavioral battery in *kat2* knockout and WT mice, and we report procedures according to ARRIVE 2.0 to maximize reproducibility. Regarding the brain samples, following the determination of tissue weights, the samples were homogenized using an ultrasonic homogenizer (UP100H, Hielscher Ultrasound Technology, Germany) set to 100% amplitude and a 0.5 cycle setting. Homogenization was performed in threefold volumes of ice-cooled LC-MS grade water relative to tissue mass (e.g., 90 µL of water was added to 30.0 mg of tissue). Data from any brain regions in which tissue preparation or homogenization did not meet predefined quality standards were excluded from subsequent analyses to ensure methodological consistency and data reliability. We quantified Trp-KYN, serotonergic, and indole metabolites, plus catecholamine intermediates and selected cofactors, using previously published multiplex LC-MS/MS methods and protocols [123,124]. For PA, the MRM transition was 124.0 → 106.0 m/z, with a retention time of 1.21 min, using a declustering potential of 75 V and a collision energy of 13 V. For ICA, the MRM transition was 146.1 → 118.0 m/z, with a retention time of 12.40 min, using a declustering potential of 50 V and a collision energy of 19 V. For IPA, the MRM transition was 190.1 → 130.1 m/z, with a retention time of 13.00 min, using a declustering potential of 50 V and a collision energy of 19 V. For ILA, the MRM transition was 206.1 → 188.1 m/z, with a retention time of 12.00 min, using a declustering potential of 50 V and a collision energy of 13 V. For INS, the MRM transition was 211.9 → 131.9 m/z, with a retention time of 11.80 min, using a declustering potential of −50 V and a collision energy of −25 V. For pCS, the MRM transition was 186.9 → 107.0 m/z, with a retention time of 12.70 min, using a declustering potential of −50 V and a collision energy of −26 V. All reagents and chemicals were of analytical or liquid chromatography–mass spectrometry grade. Trp and its metabolites, and their deuterated forms: d4-serotonin, d5-tryptophan, d4-kynurenine, d5-kynurenic acid, d4-xanthurenic acid, d5-5-hydroxyindole-acetic acid, d3-3-hydroxyanthranilic acid, d4-picolinic acid, and d3-quinolinic acid were purchased from Toronto Research Chemicals (Toronto, ON, Canada). d3-3-hydroxykynurenine was obtained from Buchem B. V. (Apeldoorn, The Netherlands). Acetonitrile (ACN) was provided by Molar Chemicals (Halásztelek, Hungary). Methanol (MeOH) was purchased from LGC Standards (Wesel, Germany). Formic acid (FA) and water were obtained from VWR Chemicals (Monroeville, PA, USA). The UHPLC-MS/MS system consisted of a PerkinElmer Flexar UHPLC system (two FX-10 binary pumps, solvent manager, autosampler, and thermostatic oven; all PerkinElmer Inc. (Waltham, MA, USA)), coupled to an AB SCIEX QTRAP 5500 MS/MS triple quadrupole mass spectrometer and controlled by Analyst 1.7.1 software (both AB Sciex, Framingham, MA, USA).

#### 2.5.2. Plasma and Urine Samples

Plasma and urine samples were collected, prepared, and measured according to previously published methodologies [67,123,124]. The samples were collected between 8 a.m. and 12 p.m. local time to limit circadian variability.

### 2.6. The Enzyme Activities of Tryptophan (Trp) Metabolism

The enzyme activity of Trp metabolism was estimated by calculating the ratio of the product-to-substrate concentration ratio.

### 2.7. Oxidative Stress and Excitotoxicity Indices

The oxidative stress index was derived by calculating the ratio between the concentration of the presumed pro-oxidant metabolite 3-HK and the combined concentrations of the putative antioxidant metabolites KYNA, anthranilic acid (AA), and xanthurenic acid (XA) (Equation (4)).

(4) $$Oxidative\;stress\;index\;=\;\frac{\left[3-hydroxykynurenine\right]}{\left[Kynurenic\;acid\right]+\left[Anthranilic\;acid\right]+[Xanthurenic\;acid]}$$

The excitotoxicity index was determined by computing the ratio of quinolinic acid (QA), an NMDA receptor agonist, to KYNA, an endogenous NMDA receptor antagonist (Equation (5)).

(5) $$Excitotoxicity\;index\;=\;\frac{\left[Quinolinic\;acid\right]}{\left[Kynurenic\;acid\right]}$$

### 2.8. Statistical Analysis

All statistical analyses were conducted using IBM SPSS Statistics, version 28.0.0.0 (IBM Corp., Armonk, NY, USA). The normality of data distribution was assessed with the Shapiro–Wilk test, and Q-Q plots were additionally employed to evaluate whether two datasets originated from the same distribution.

In the statistical evaluation of the NORT, OBAT, and Y-maze, 3CT, and rotarod test, inter-strain comparisons were performed using the independent samples *t*-test for normally distributed data, whereas the non-parametric Mann–Whitney U test was employed in cases where the assumption of normality was violated. Intra-strain comparisons of individual parameters were carried out using the paired samples *t*-test when data conformed to a normal distribution, and the Wilcoxon signed-rank test was applied for non-normally distributed datasets.

For the MBT, a mixed ANOVA model was used, followed by the Tamhane post hoc test.

Regarding the UHPLC-MS/MS measurements, the normality of the variables was checked using the Kolmogorov–Smirnov test and visually checked using quantile-quantile plots, and the equality of variances was examined using Welch’s F-test. Outliers were identified using Grubbs’s test. Comparisons between the two groups were conducted using an independent samples *t*-test.

Values *p* < 0.05 were considered statistically significant. Our data are reported as means ± standard deviations (SD) for all parameters and experimental groups.

## 3. Results

### 3.1. Behavioral Tests

#### 3.1.1. Novel Object Recognition Test (NORT)

**Figure 2:**
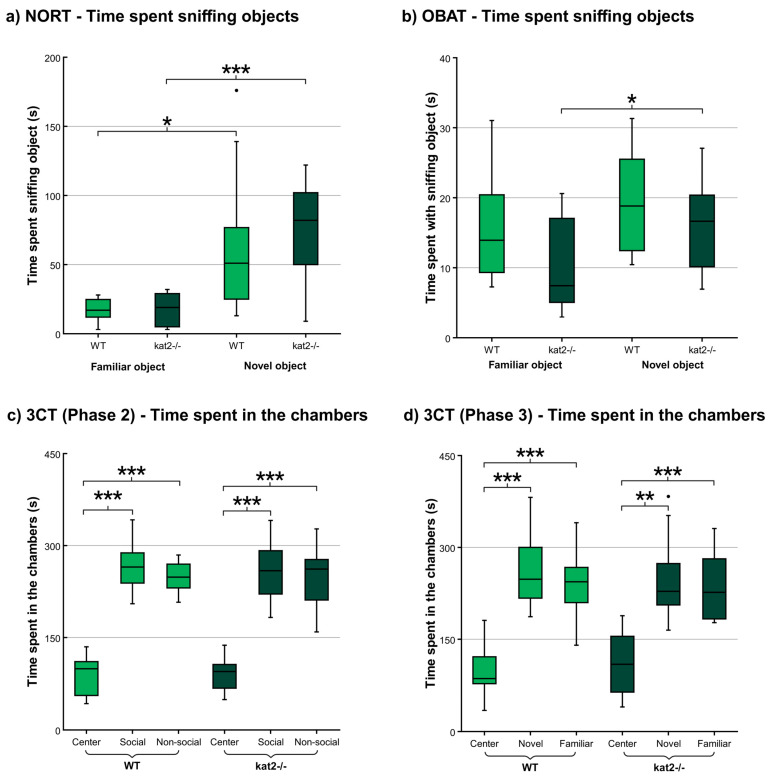
Behavioral assessment of wild-type (WT) and *kat2^−^/^−^* mice in object recognition and social interaction paradigms. (**a**) Time spent sniffing familiar vs. novel objects in the novel object recognition test (NORT). During the NORT, both WT and *kat2^−/−^* animals spent significantly more time exploring the novel object. (**b**) Time spent sniffing familiar vs. novel objects in the object-based attention test (OBAT). In the OBAT, the mutant strain spent more time with the novel object. (**c**) Time spent in the center, social, and non-social chambers during the three-chamber test (3CT, Phase 2). Both WT and *kat2^−/−^* mice spent more time in the side chambers than in the center chamber. (**d**) Time spent in the center, novel animals, and familiar animals’ chambers during the 3CT (Phase 3). Both WT and *kat2^−/−^* mice spent more time in the side chambers than in the center chamber. Wild-type mice (light green); *kat2^−^/^−^* mice (dark green). Data are presented as mean ± SD. ●, outlier. *, *p* < 0.05; **, *p* < 0.01; ***, *p* < 0.001. The figure was created with LabPlot 2.9.0 (KDE, Berlin, Germany) and BioRender.com. 3CT, three-chamber test; *kat2^−/−^*, kynurenine aminotransferase II knockout mice; NORT, novel object recognition test; OBAT, object-based attention test; WT, wild-type mice.

During the NORT, both WT and *kat2^−/−^* animals spent significantly more time interacting with the novel object compared to the familiar one. However, no significant differences were detected between the strains (Figure 2, Tables S1 and S2).

#### 3.1.2. Object-Based Attention Test (OBAT)

In the OBAT, no statistically significant differences were detected between the strains in terms of overall object interaction time. Nonetheless, animals from the *kat2^−/−^* strain exhibited a marked preference for the novel object, spending significantly more time engaging with it compared to the familiar object during the testing phase (Figure 2, Tables S1 and S2).

#### 3.1.3. Three Chamber Test (3CT)

During the 3CT, both examined strains spent significantly less time in the lateral chambers compared to the central starting chamber in both the second and third phases of the test. Apart from this difference, no other significant variations were observed (Figure 2, Tables S1 and S2).

#### 3.1.4. Other Behavioral Tests

No significant differences were observed between the strains or within strains in the Y-maze, MBT, and rotarod tests (Table S1).

### 3.2. Ultra-High-Performance Liquid Chromatography with Tandem Mass Spectrometry (UHPLC-MS/MS)

**Figure 3:**
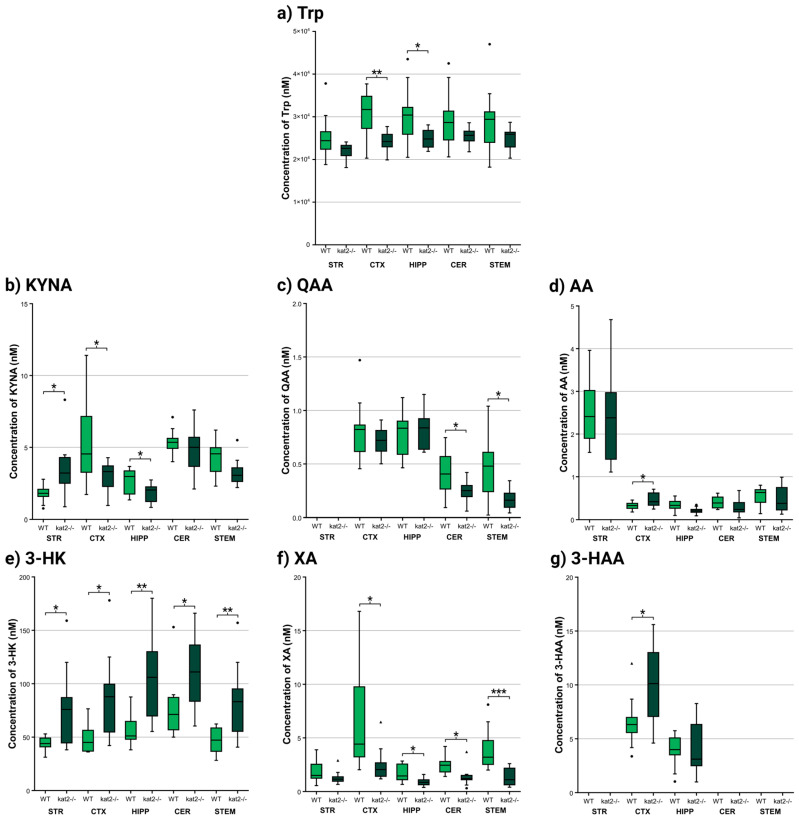
Regional distribution of kynurenine (KYN) pathway metabolites in wild-type (WT) and *kat2^−/−^* mouse brains. (**a**) Tryptophan (Trp), (**b**) Kynurenic acid (KYNA), (**c**) Quinaldic acid (QAA), (**d**) Anthranilic acid (AA), (**e**) 3-hydroxykynurenine (3-HK), (**f**) Xanthurenic acid (XA), and (**g**) 3-Hydroxyanthranilic acid (3-HAA) concentrations measured in striatum (STR), cortex (CTX), hippocampus (HIPP), cerebellum (CER), and brainstem (STEM). Trp was significantly lower in STR, CTX, and HIPP. While KYNA increased in STR, its concentration lowered in CTX and HIPP. QAA’s concentration was significantly lower in CER and STEM. The level of AA was higher in CTX. The concentration of 3-HK increased in every brain region. XA decreased in CTX, HIPP, CER, and STEM. The level of 3-HAA increased in CTX. Wild-type (WT, light green) and *kat2^−^/^−^* (dark green) groups are shown. Data are expressed as mean ± SD. ●, outlier; ▲, far out. *, *p* < 0.05; **, *p* < 0.01; ***, *p* < 0.001. The figure was created with LabPlot 2.9.0 (KDE, Berlin, Germany) and BioRender.com. 3-HAA, 3-hydroxyanthranilic acid; 3-HK, 3-hydroxykynurenine; AA, anthranilic acid; CER, cerebellum; CTX, cortex; HIPP, hippocampus; KYNA, kynurenic acid; QAA, quinaldic acid; STEM, brainstem; STR, striatum; Trp, tryptophan; WT, wild-type mice; *kat2^−^/^−^*, kynurenine aminotransferase II knockout mice; XA, xanthurenic acid.

**Figure 4:**
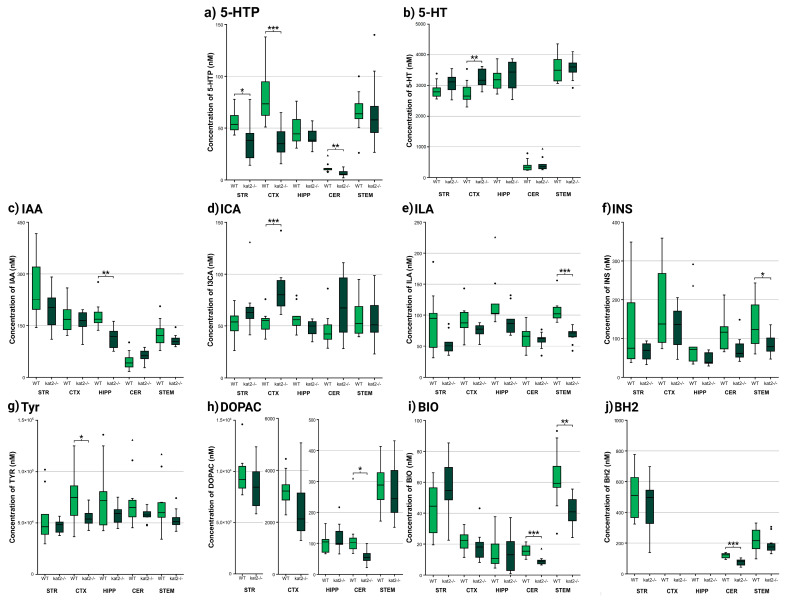
Region-specific alterations in serotonin (5-HT)-, indole-pyruvate-, and tyrosine (Tyr)–dopamine (DA)-derived metabolites in wild-type (WT) and *kat2^−^/^−^* mice. Box plots showing concentrations of metabolites across brain regions in wild-type (WT, light green) and *kat2^−^/^−^* (dark green) mice. (**a**,**b**) Serotonin pathway: 5-HTP (5-hydroxytryptophan), 5-HT (5-hydroxytryptamine, serotonin). (**c**–**f**) Indole-pyruvate pathway: IAA (indole-3-acetic acid), ICA (indole-3-carboxaldehyde), ILA (indole-3-lactic acid), INS (indoxyl sulfate). (**g**–**j**) Tyrosine-dopamine pathway: Tyr, DOPAC (3,4-dihydroxyphenylacetic acid), BIO (biopterin), BH2 (dihydrobiopterin). Brain regions: STR, striatum; CTX, cortex; HIPP, hippocampus; CER, cerebellum; STEM, brainstem. 5-HTP concentrations were reduced in the STR, CTX, and CER, whereas 5-HT levels were selectively increased in the CTX. IAA concentrations were diminished in the HIPP, ILA, and INS within the STEM, while ICA levels were elevated in the CTX. Tyr levels were reduced in the CTX, and decreases in DOPAC, BIO, and BH2 were detected in the CER. Data are shown as mean ± SD. Symbols: •, outlier; ▲, far out. Significance: *, *p* < 0.05; **, *p* < 0.01; ***, *p* < 0.001. Figures created with LabPlot 2.9.0 (KDE, Berlin, Germany) and BioRender.com. 5-HT, serotonin (5-hydroxytryptamine); 5-HTP, 5-hydroxytryptophan; BH2, dihydrobiopterin; BIO, biopterin; CER, cerebellum; CTX, cortex; DOPAC, 3,4-dihydroxyphenylacetic acid; HIPP, hippocampus; ICA, indole-3-carboxaldehyde; IAA, indole-3-acetic acid; ILA, indole-3-lactic acid; INS, indoxyl sulfate; *kat2*^−/−^, kynurenine aminotransferase II knockout mice; STEM, brainstem; STR, striatum; Tyr, tyrosine; WT, wild-type mice.

**Figure 5:**
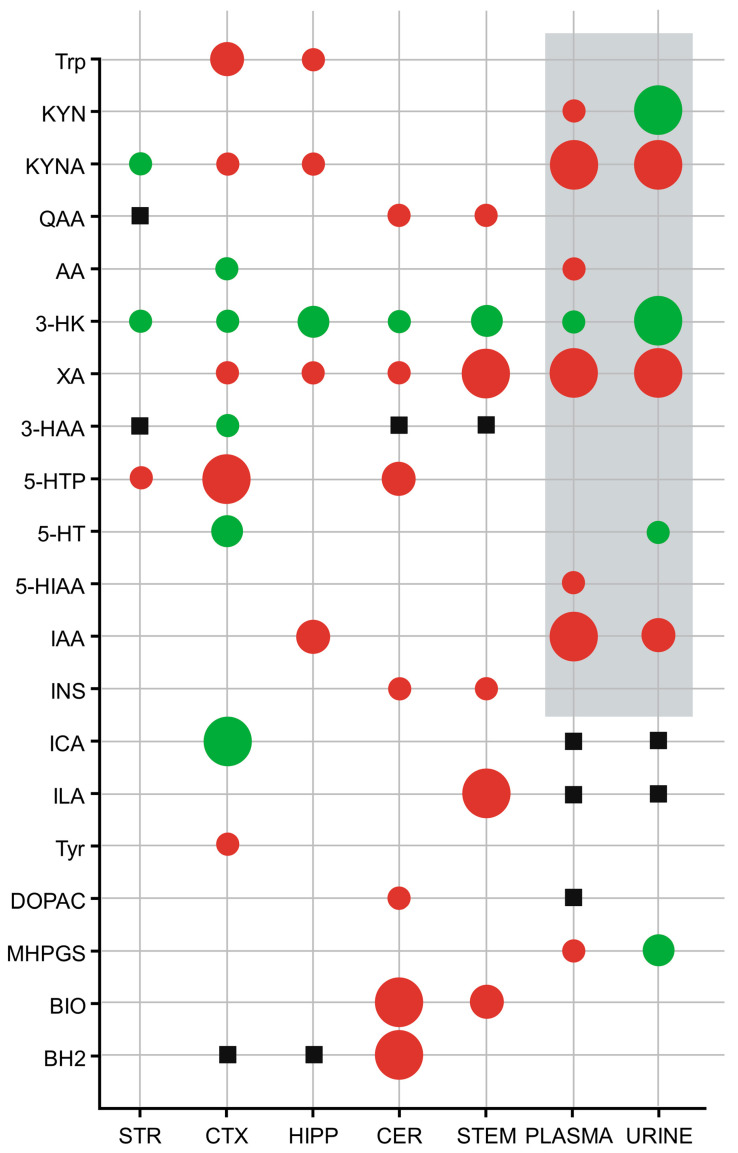
Overview of region- and matrix-specific alterations in tryptophan-derived metabolites in 8-week-old male *kat2^−/−^* mice compared to wild-type (WT) controls in ultra-high-plasticity liquid chromatography with tandem mass spectrometry. This figure summarizes significant alterations in tryptophan–kynurenine, serotonin, indole-pyruvate, and tyrosine–dopamine pathway metabolites across distinct brain regions (striatum, cortex, hippocampus, cerebellum, brainstem), plasma, and urine in *kat2^−/−^* mice compared to the WT. Results highlight the region- and pathway-selective metabolic rewiring induced by *kat2* deletion, particularly the consistent increase of 3-hydroxykynurenine and decrease in xanthurenic acid, alongside mixed kynurenic acid responses and downstream shifts in serotonin, indole, and catecholamine derivatives. We marked significant changes with circles. Red circles mean a statistically significant decrease, and green shows a significant increase in the concentration compared to the WT mice. The increasing size of the circles indicates higher levels of significance (small circle: *p* < 0.05; medium circle: *p* < 0.01; large circle: *p* < 0.001). Gray rectangle background: previously published results [67]. Black square: no data. 3-HAA, 3-hydroxyanthranilic acid; 3-HK, 3-hydroxykynurenine; 5-HIAA, 5-hydroxyindole-3-acetic acid; 5-HT, serotonin (5-hydroxytryptamine); 5-HTP, 5-hydroxytryptophan; AA, anthranilic acid; BH2, dihydroxybiopterin; BIO, biopterin; CER, cerebellum; CTX, cortex; DOPAC, 3,4-dihydroxyphenylacetic acid; HIPP, hippocampus; IAA, indole acetic acid; ICA, indole-3-carboxaldehyde; ILA, indole-3-lactic acid; INS, indoxyl sulfate; KYN, kynurenine; KYNA, kynurenic acid; MHPGS, 3-methoxy-4-hydroxyphenylglycol sulfate; QAA, quinaldic acid; Trp, tryptophan; Tyr, tyrosine; STEM, brainstem; STR, striatum; XA, xanthurenic acid.

Several differences were observed between the WT and mutant strains in the various brain regions examined during the chemical analytical measurements (Figure 3, Figure 4 and Figure 5, Tables S3 and S4). The most prominent difference was observed in the level of 3-HK, which was uniformly and significantly increased in all examined brain regions of the *kat2^−/−^* strain compared to the WT. In a similar pattern, xanthurenic acid (XA) levels were reduced across all analyzed regions relative to the WT. In contrast, the concentration of KYNA, whose alteration was most strongly anticipated, exhibited a significant reduction only in the CTX and HIPP; however, the level of KYNA is increased in the STR. Interestingly, the level of its downstream metabolite, quinaldic acid (QAA), remained unaltered in these same areas, while a marked decrease was observed in the CRB and STEM.

In addition to the detailed concentration values presented above, a region- and matrix-integrated overview of metabolite alterations is provided (Table S3). Consistent across all examined brain regions, 3-HK was significantly elevated, whereas XA showed a uniform reduction, underscoring a shift toward an oxidative and excitotoxic milieu. KYNA exhibited a divergent pattern, being decreased in the CTX and HIPP yet elevated in the STR, while QAA was selectively reduced in the CER and STEM. Within the serotonergic pathway, 5-hydroxytryptophan (5-HTP) declined in CTX and CER, whereas 5-HT increased in CTX and urine, indicating altered turnover. Further changes included decreased Tyrin CTX and HIPP and selective reductions in pterins. Peripheral findings in plasma and urine, previously published, are integrated here for comparison, emphasizing the concordance between central and systemic metabolic rewiring.

Complementing our previous measurements of Trp metabolite levels in plasma and urine, we also measured the concentrations of additional metabolites from the indole-pyruvate and TYR-DA pathways; however, we observed significant changes only in 3-methoxy-4-hydroxyphenylglycol sulfate (MHPGS) levels compared to WT (Figure 5, Table S4).

### 3.3. Enzyme Activities

**Figure 6:**
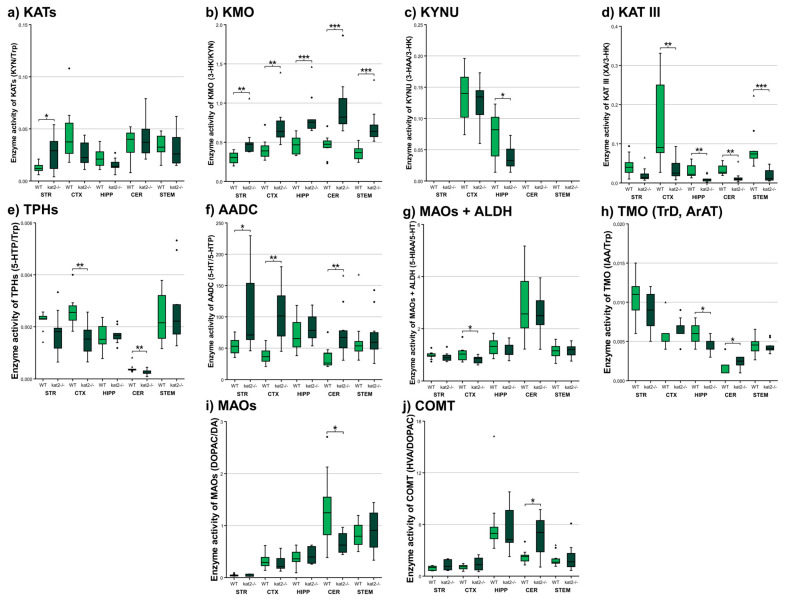
Regional enzyme activity alterations in tryptophan- and serotonin (5-HT)-associated pathways in WT and *kat2^−/−^* mice. Box plots showing enzyme activities across striatum (STR), cortex (CTX), hippocampus (HIPP), cerebellum (CER), and brainstem (STEM) in wild-type (WT, light green) and *kat2^−/−^* (dark green) mice. Activities were calculated as product-to-substrate ratios for: (**a**) KATs (KYNA/KYN), (**b**) KMO (3-HK/KYN), (**c**) KYNU (3-HAA/3-HK), (**d**) KAT III (XA/3-HK), (**e**) TPHs (5-HTP/Trp), (**f**) AADC (5-HT/5-HTP), (**g**) MAOs + ALDH (5-HIAA/5-HT), (**h**) TMO [TrD, ArAT] (IAA/Trp), (**i**) MAOs (DOPAC/DA), (**j**) COMT (HVA/DOPAC). Enzyme activity of KATs decreased in STR. KMO’s activity significantly increased in every brain region. KYNU’s activity decreased in HIPP. KAT III enzyme activity decreased in CTX, HIPP, CER, and STEM. Activity of TPH enzymes decreased in CTX and CER. AADC’s activity decreased in STR, CTX, and CER. MAOs + ALDH decreased in CTX. TMO’s activity decreased in HIPP and CER. Activity of MAOs in the tyrosine-dopamine pathway significantly decreased in CER. COMT’s activity increased in CER. Data are shown as mean ± SD. Symbols: •, outlier; ▲, far out. Statistical significance: *, *p* < 0.05; **, *p* < 0.01; ***, *p* < 0.001. Figures were created with LabPlot 2.9.0 (KDE, Berlin, Germany) and BioRender.com. 3-HAA, 3-hydroxyanthranilic acid; 3-HK, 3-hydroxykynurenine; 5-HIAA, 5-hydroxyindoleacetic acid; 5-HT, serotonin (5-hydroxytryptamine); 5-HTP, 5-hydroxytryptophan; AADC, aromatic L-amino acid decarboxylase; ALDH, aldehyde dehydrogenase; ArAT, aromatic amino acid aminotransferase; CER, cerebellum; COMT, catechol-O-methyltransferase; CTX, cortex; DA, dopamine; DOPAC, 3,4-dihydroxyphenylacetic acid; HIPP, hippocampus; HVA, homovanillic acid; IAA, indole-3-acetic acid; KAT III, kynurenine aminotransferase III; KATs, kynurenine aminotransferases; *kat2^−/−^*, kynurenine aminotransferase II knockout; KMO, kynurenine-3-monooxygenase; KYN, kynurenine; KYNU, kynureninase; MAOs, monoamine oxidases; STEM, brainstem; STR, striatum; TMO, tryptophan-2-monooxygenase; TPHs, tryptophan hydroxylases; TrD, tryptophan decarboxylase; Trp, tryptophan; WT, wild-type mice; XA, xanthurenic acid.

Enzyme activity ratios revealed pronounced remodeling of tryptophan metabolic fluxes in *kat2^−/−^* mice (Figure 6, Table S5). As expected, KAT activity (KYN/Trp) displayed a selective reduction in the STR, consistent with the genetic deletion of KAT II. Conversely, KMO activity (3-HK/KYN) was markedly elevated across STR, CTX, HIPP, CER, and STEM, indicating enhanced pro-oxidant pressure through 3-HK production. KYNU activity (3-HAA/3-HK) showed a modest yet significant increase in the HIPP, while KAT III activity (XA/3-HK) was consistently reduced in CTX, HIPP, CER, and STEM, reinforcing the loss of protective XA formation. Within the serotonergic arm, tryptophan hydroxylase (TPH) activity (5-HTP/Trp) was reduced in CTX and CER, whereas aromatic L-amino acid decarboxylase (AADC) activity (5-HT/5-HTP) was elevated in STR, CTX, and HIPP, suggesting compensatory 5-HT turnover. MAO/ALDH activity (5-HIAA/5-HT) was decreased only in CTX, while TMO activity (IAA/Trp) was reduced in CTX, HIPP, and CER. Furthermore, the activity of MAOs (DOPAC/DA) significantly decreased, while the activity of COMT (HVA/DOPAC) increased in CER. Collectively, these shifts highlight a pathway-specific reorganization, with KMO dominance, curtailed XA buffering, and altered 5-HT–indole and Tyr-DA dynamics shaping the *kat2^−/−^* metabolic phenotype.

### 3.4. Oxidative Stress and Ecitotoxicity Indices

**Figure 7:**
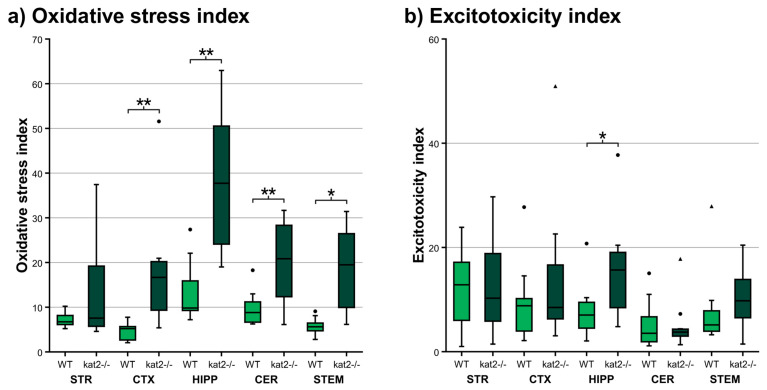
Regional indices of oxidative stress and excitotoxicity in wild-type (WT) and *kat2^−/−^* mice. Box plots showing oxidative stress and excitotoxicity indices across striatum (STR), cortex (CTX), hippocampus (HIPP), cerebellum (CER), and brainstem (STEM) in WT (light green) and *kat2^−/−^* (dark green) mice. (**a**) Oxidative stress index, calculated as the ratio 3-HK/(KYNA + AA + XA), reflecting the balance between pro-oxidant and antioxidant metabolites. The oxidative stress index significantly increased in CTX, HIPP, CER, and STEM. (**b**) Excitotoxicity index, calculated as the ratio QA/KYNA, representing the N-methyl-D-aspartate (NMDA) receptor agonist-to-antagonist balance. The excitotoxicity index increased in HIPP. Data are shown as mean ± SD. Symbols: •, outlier; ▲, far out. Statistical significance: *, *p* < 0.05; **, *p* < 0.01. Figures generated with LabPlot 2.9.0 (KDE, Berlin, Germany) and BioRender.com. 3-HK, 3-hydroxykynurenine; AA, anthranilic acid; CER, cerebellum; CTX, cortex; HIPP, hippocampus; *kat2^−/−^*, kynurenine aminotransferase II knockout mice; KYNA, kynurenic acid; QA, quinolinic acid; STEM, brainstem; STR, striatum; WT, wild-type mice; XA, xanthurenic acid.

To further evaluate the balance between oxidative pressure and excitotoxic potential, we calculated composite indices from key KYN metabolites (Figure 7, Table S6). The oxidative stress index, defined as 3-HK/(KYNA + AA + XA), was significantly elevated in multiple brain regions of *kat2^−/−^* mice. Compared to WT controls, the CTX, HIPP, CER, and STEM all exhibited marked increases, with the HIPP and STEM showing the most robust elevations. This pattern reflects the combined impact of increased 3-HK and reduced antioxidant metabolites, underscoring a shift toward pro-oxidant load. In contrast, the excitotoxicity index, measured as QA/KYNA, showed a more selective profile. While values remained unchanged in STR, CTX, and CER, a significant increase emerged in the HIPP, where diminished KYNA coincided with elevated QA. A similar trend, though nonsignificant, was observed in the STEM. Taken together, these data indicate that KAT II deficiency imposes a dual burden of oxidative stress and region-specific excitotoxic vulnerability, with the HIPP emerging as a particularly sensitive locus of metabolic imbalance.

Results begin with region-resolved metabolomics across STR, CTX, HIPP, CER, and STEM, followed by enzyme-ratio proxies, cofactor mapping, and alignment with behavioral outcomes. A convergent central signature emerges: 3-HK increases and XA decreases pan-regionally, KYNA reduction localizes to CTX and HIPP, KMO activity indices rise while KAT III flux falls, and oxidative-stress measures increase broadly, with a hippocampal-specific rise in the excitotoxicity index. Cofactor analyses identify BIO depletion in CER and STEM and BH2 loss in CER. Gut–brain and serotonergic markers also shift, with hippocampal IAA reduction, cortical and cerebellar ICA elevation, and reduced cortical 5-HT turnover, yet cognition, sociability, and coordination remain intact. While these preserved functions are reported alongside metabolic profiles, we did not conduct formal correlations linking regional metabolite concentrations to behavioral readouts. This omission reflects the study scope and sample size rather than a conceptual barrier. We now state this as a limitation and outline plans to evaluate specific associations, such as hippocampal KYNA with NORT performance, within a prospectively powered framework. Consistent with region-specific rewiring rather than a uniform KAT II effect, we note a paradoxical pattern: striatal KYNA elevation likely reflects compensatory KAT isoforms or astrocyte-mediated KYN shunting; forebrain bias toward AA aligns with oxidative-stress–driven KMO→KYNU flux that diverts 3-HK from KYNA. In sum, findings delineate a KMO-tilted, pterin-constrained, indole-modulated milieu that selectively burdens affective and motor subdomains while sparing core cognitive and social functions.

## 4. Discussion

The metabolism of Trp has emerged as a central hub in the neurobiology of cognition, mood, and social behavior, with imbalances along its KYN, 5-HT, indole, and DA branches increasingly linked to neuropsychiatric and neurodegenerative disorders [15,16,22,28,39]. Within this network, KAT II has been considered the dominant enzymatic source of KYNA, a metabolite long viewed as neuroprotective through NMDA, α7-nAChR, AMPA, and kainate antagonism [20,67,125,126,127,128,129,130]. Yet, earlier reports of global KAT II inhibition or genetic deletion left unresolved how local shifts in KYNA and related metabolites shape functional brain states [20,28,67,126,129,131]. By combining region-resolved metabolomics with behavioral phenotyping, our study addresses this gap, asking whether neurochemical disequilibria in KAT II deficiency necessarily translate into overt cognitive, motor, or social dysfunction [20,22,67,132,133]. To support cross-domain reading, behavioral tasks are mapped to region-specific metabolite indices using shared labels and synchronized panel order, pairing NORT and three chambers with cortical and hippocampal KYNA and 3 HK, and Rotarod with striatal metrics.

Mechanistic bridge linking regional rewiring to behavior. The metabolomic pattern combines KYNA reduction in CTX and HIPP with KYNA elevation in STR, alongside higher oxidative pressure in several regions and a hippocampal rise in the excitotoxicity index. Such spatial heterogeneity can stabilize baseline outputs via compensatory gating while lowering the threshold for deficits when tasks recruit prefrontal hippocampal integration or impose stress. This perspective shifts the focus from global impairment to domain-specific resilience versus vulnerability that depends on region and task demand.

Notably, the lack of inter-strain differences across cognitive, social, and motor tests suggests that *kat2* deletion does not inherently induce depressive-like behavior under baseline conditions. This finding contrasts with our previous report of despair-linked phenotypes under stress paradigms [67], implying that the emotional alterations in *kat2^−^/^−^* mice are context-dependent. Rather than manifesting as overt depression-like behavior, the current data support a model in which KAT II deficiency biases affective circuitry through neurochemical disequilibrium, particularly elevated 3-HK and diminished KYNA, thereby establishing a stress-reactive emotional predisposition. Hence, “emotional bias” in this context refers to a neurochemical vulnerability rather than a direct behavioral outcome.

The metabolic profile emerging from *kat2^−/−^* brains reveals a striking shift toward a pro-oxidant milieu, characterized by pan-regional accumulation of 3-HK and consistent loss of XA, with an additional region-selective reduction in KYNA in CTX and HIPP. Such changes converge on a biochemical signature that indicates heightened KMO activity alongside impaired KAT II flux, effectively tilting the KYN pathway toward neurotoxic branch products [35,67,134]. The imbalance between reduced antioxidant buffering (XA, KYNA) and sustained excitatory drive amplifies oxidative-stress indices across regions, with the HIPP exhibiting a peak excitotoxicity signal. This constellation suggests that, although behavioral performance remained intact, the HIPP in particular resides in a precarious metabolic state, vulnerable to secondary insults [135]. Thus, the observed disequilibrium highlights a latent central risk architecture, in which KAT II deficiency may predispose selective circuits to degeneration under stress or aging.

The selective decline of KYNA in HIPP and CTX is particularly consequential, as these structures form the backbone of glutamatergic integration underlying memory, attentional control, and affective regulation [126,136,137,138,139,140]. Lower KYNA levels in these regions imply diminished tonic antagonism at NMDA and α7-nicotinic receptors, a state that can facilitate plasticity yet simultaneously heighten vulnerability to excitotoxic cascades. Such changes resonate with the well-documented link between KYN pathway imbalance and affective or cognitive disturbances. In contrast, the cerebellar and STEM pattern, where QAA and related metabolites decline, speaks to circuits subserving motor coordination and arousal [126,136,137,141]. Here, altered metabolic buffering could subtly recalibrate sensorimotor integration and vigilance states, aligning with CER–STEM contributions to motor timing and autonomic tone. Together, these region-specific shifts underscore circuit-level rebalancing rather than global disruption.

The consistent elevation of 3-HK across regions, paralleled by a decline in XA, underscores a shift toward a redox-imbalanced milieu that favors oxidative stress [142,143,144]. This pro-oxidant tilt is further amplified when considered in the context of derived indices, where the 3-HK–to–antioxidant ratio signals a vulnerability state rather than an immediate injury [142,143,144]. In parallel, the excitotoxicity index, weighted by QA/KYNA balance, delineates selective windows in which NMDA drive may outweigh intrinsic antagonism [140,142,143,145,146]. These biochemical loads, however, need not manifest uniformly as behavioral deficits at baseline [147,148,149]. Accordingly, references to depression or post-traumatic stress disorder denote hypothesis-generating convergence on shared metabolic nodes, not confirmation of disorder-specific phenotypes in mice [67,150,151]. Instead, they may represent latent liabilities, poised to surface under developmental, aging, or environmental stressors, thereby marking a hidden susceptibility rather than an overt phenotype [15,143,144].

Region-specific perturbations in the pterin pool highlight a subtle but consequential layer of metabolic vulnerability [152,153,154]. In *kat2^−/−^* hindbrain, the concurrent depletion of BIO and loss of BH_2_ suggest an erosion of the redox-cycling capacity that normally safeguards BH_4_ availability [152,153,154]. Because BH_4_ is indispensable for tyrosine hydroxylase activity, even modest shifts in this cofactor equilibrium may attenuate DA biosynthesis and, secondarily, compromise broader monoaminergic tone [152,153,154,155]. These alterations could reverberate into nitric oxide biology, given that endothelial and neuronal nitric oxide synthase (NOS) also require BH_4_, thereby coupling monoamine insufficiency to redox imbalance and impaired vasomodulation [152,153,154].

The neurochemical profile in *kat2^−/−^* mice suggests that reduced serotonergic turnover in the CTX converges with decreased hippocampal IAA and concomitant elevations of ICA in cortical and cerebellar regions to shape circuit-level excitability [156,157,158]. Lower 5-HT turnover may weaken cortical inhibitory tone, thereby amplifying the impact of indole-derived signaling [157,158]. The reduction in IAA, a ligand with protective barrier and anti-inflammatory properties, contrasts with the elevation of ICA, a potent AhR agonist capable of reprogramming microglial states [156,157,158]. Through this shift, cortical and cerebellar microglia may adopt transcriptional phenotypes that subtly recalibrate glutamatergic drive and synaptic responsiveness [156,157,158]. Such AhR-mediated modulation, in concert with altered serotonergic dynamics, delineates a mechanism by which KAT II deficiency reshapes microcircuit stability without overtly impairing cognitive or social behaviors.

The parallel remodeling of Trp metabolism across central and peripheral compartments suggests a degree of concordance that greatly enhances translational traction [15,159,160]. When brain signatures mirror those detected in plasma or urine—such as the uniform elevation of 3-HK and the consistent reduction in XA—biomarker feasibility is strengthened because measurements in accessible fluids reliably report on neurochemical states [15,159,160]. This concordance further supports longitudinal monitoring, since repeated peripheral sampling can index dynamic shifts in pathway fluxes without invasive procedures [15,159,160]. Importantly, the pattern of a KMO-tilted, pterin-constrained phenotype provides a stratification handle: individuals or models exhibiting this profile may constitute a distinct subgroup marked by heightened oxidative burden and diminished neuroprotection [15,131,159]. Thus, the central–peripheral alignment not only validates observed signatures but also operationalizes them for precision tracking and targeted intervention [161].

Despite a pervasive biochemical tilt toward oxidative stress and excitotoxic vulnerability, baseline cognition and sociability remain largely preserved in *kat2^−/−^* mice. This dissociation likely reflects both redundancy within cognitive and social circuits and compensatory plasticity that stabilizes performance until a higher stress threshold is breached [67,162,163]. Cortico-hippocampal KYNA loss may sensitize affective and attentional pathways, but parallel buffering via dopaminergic and indole-linked mechanisms appears sufficient to maintain recognition memory and sociability in standard tasks. In contrast, motor and affective domains, subserved by CER–STEM loops and stress-responsive hippocampal circuits, manifest early pressure, consistent with lower redundancy and higher task sensitivity. These findings underscore that metabolic disequilibria need not uniformly generalize to behavior and that endpoint detectability hinges on domain-specific thresholds. For experimental design, this argues against relying solely on cognition- or sociability-based readouts and instead favors composite panels that capture latent affective or motor liabilities, especially when probing therapeutic interventions or stress challenges.

The paradoxical distribution of metabolites across brain regions points to a region-specific metabolic rewiring rather than a uniform effect of *kat2* loss [125,164]. Striatal KYNA increased, while it declined in CTX and HIPP and remained unchanged in CER and STEM. This striatal KYNA elevation in the absence of *kat2* likely arises from alternative KAT isoform activity or astrocyte-mediated KYN shunting, consistent with the region’s dense dopaminergic and glial milieu [165]. In contrast, plasma and urine showed marked KYNA reductions, underscoring that regional enzymatic compensation and astrocytic buffering, rather than systemic availability, govern KYN metabolism within the brain.

Serotonergic metabolism revealed equally complex shifts. 5-HTP fell consistently in STR, CTX, and CER, suggesting precursor depletion, yet cortical 5-HT paradoxically rose. This contrast hints at selective upregulation of decarboxylase activity or altered transporter dynamics in cortical circuits. Importantly, urine samples captured a 5-HT increase, while plasma remained unaltered—underscoring a clear brain–periphery mismatch. Similarly, Trp declined in CTX and HIPP but stayed unchanged peripherally, whereas KYN itself was stable in the brain yet shifted downward in plasma and upward in urine.

Other metabolites highlighted both paradoxical and stable nodes. 3-HK rose broadly across central and peripheral compartments, but its downstream metabolite XA decreased everywhere, revealing a systemic enzymatic bottleneck. AA, however, increased in CTX but decreased in HIPP and STEM, with no peripheral reflection. Several intermediates, including Tyr and 3-HAA, remained stable in most regions, pointing to strong compensatory buffering. Taken together, these findings illustrate that *kat2* deletion drives a patchwork of paradoxical imbalances, where regional demands, oxidative stress, and glial density dictate divergent metabolic trajectories, while peripheral readouts capture only a partial, homogenized snapshot of these changes.

Furthermore, the single-gene *kat2* knockout, which markedly reduces KYNA concentrations, exerts consequences that extend far beyond the KYN branch alone. The disruption alters the enzymatic dynamics and metabolite distribution of the Trp–KYN axis and also propagates into parallel domains, such as serotonin biosynthesis, indole-pyruvate flux, and DA turnover. These cross-pathway perturbations likely emerge from shared substrate dependencies and competitive enzymatic hierarchies, wherein the depletion of one metabolic sink amplifies pressure on adjacent routes. In particular, diminished serotonin availability can be interpreted as a direct consequence of altered Trp partitioning, whereas DA irregularities appear linked to secondary changes in redox balance and cofactor utilization. Thus, *kat2* deletion should not be conceptualized merely as a KYNA-specific deficit; rather, it reshapes a broader neurochemical network in which serotonergic, dopaminergic, and indole-derived pathways become entrained into a cascade of adaptive yet destabilizing responses.

A key strength of this study lies in the methodological rigor that enabled reliable mapping of subtle, region-specific metabolic shifts. The UHPLC-MS/MS workflow was not only optimized for brain tissue matrices but was applied in a region-resolved fashion, thereby allowing contrasts across CTX, HIPP, STR, CER, and STEM rather than relying on pooled homogenates [166,167,168]. Enzyme activities were inferred through calibrated product–substrate ratios, providing functional proxies that extend beyond absolute metabolite abundance [166,168,169]. Importantly, these measurements were embedded in a multi-axis coverage that integrated KYN, serotonergic, indole-derived, and catecholamine pathways. Behavioral assays were performed with a balanced panel that minimizes habituation or training artifacts. Such a design requires a composite technical skill set—ranging from advanced metabolomics and stringent quality control pipelines to expertise in behavioral neuroscience and translational modeling—ensuring robust and reproducible interpretation.

Several limitations should be recognized when interpreting these findings. First, the study design relied on baseline-only testing, which restricts inference on developmental trajectories or dynamic responses to stressors [170,171,172]. Bulk tissue homogenates were analyzed, inevitably averaging across heterogeneous cell types and masking circuit-specific alterations [170,172,173]. The absence of cell-type resolution is particularly relevant, as astrocytic and neuronal pools may contribute divergently to KYN pathway flux [170,172,173]. Temporal resolution was also limited to a single time point, precluding assessment of circadian phase–dependent or activity-driven fluctuations [170,172,174]. Sex and age effects remain underpowered, raising the possibility that strain differences may emerge in females or in older cohorts [170,172,173]. An important limitation is the absence of microbiome profiling. The microbiome was not systematically characterized, despite its capacity to shape Trp-indole metabolism [175,176,177]. To establish intestinal origin and systemic distribution, future work will pair brain measurements with quantification of indole derivatives in fecal and plasma samples. This paired design will allow direct assessment of compartmental gradients and transport [178,179,180]. Without taxonomic or functional data, shifts in ICA, IAA, and INS cannot be assigned to specific microbial pathways or sources, which narrows mechanistic inference regarding gut–brain communication in this dataset [175,176,177]. To dissect source contributions, we will consider microbiota-targeted interventions, including antibiotic depletion, fecal microbiota transplantation, and probiotic supplementation. These perturbations, combined with fecal and plasma profiling of ICA, IAA, and related indoles, can distinguish host from microbial activity and probe their interaction, thereby sharpening translational relevance [181,182,183]. Finally, circadian control was standardized but not manipulated, and phase-dependent metabolic reorganization could shift interpretations [170,172,174].

The neurochemical profile observed in *kat2^−/−^* mice points toward several therapeutic avenues [67,129,143,184]. Elevated 3-HK alongside reduced XA underscores excessive KMO flux, highlighting selective KMO inhibition as a rational intervention to rebalance the neuroprotective–neurotoxic equilibrium [184,185]. At the same time, the consistent depletion of BH4 and riboflavin-sensitive cofactor pools suggests that restoring the pterin milieu could stabilize DA synthesis and restrain aberrant redox cycling [129,184]. Antioxidant strategies targeting the 3-HK–driven oxidative load may provide additional neuroprotection, particularly in regions where KYNA is diminished [55,129]. Importantly, our data emphasize the need for composite biomarker frameworks that integrate peripheral indices of KYN pathway activity with region-sensitive readouts such as KYNA/QAA ratios, thereby offering translational precision in stratifying patients [55,67,161,184]. For navigation, Table 1 crosswalks behavioral endpoints with regional metabolite and cofactor panels using shared labels and coordinated panel order.

Microbiota-derived indoles may also function as behavior modulators through AhR-dependent signaling, glial state regulation, and serotonergic control of network excitability [156,186,187]. Aligning indole panels with selected behavioral readouts can link peripheral variation to circuit-level outcomes while maintaining conservative inference [52,188]. These convergent insights open the path toward mechanism-guided interventions that cut across psychiatric and neurodegenerative spectra [55,67,184]. Accordingly, microbiota-targeted designs should prespecify behavioral endpoints sensitive to indole tone, including NORT discrimination, three-chamber novelty preference, and open-field indices [161]. Such alignment enables tests of correspondence between ICA, IAA, or INS shifts and measurable changes in performance without reinterpreting existing results [52,189,190]. Furthermore, future work should integrate stool metagenomics or metatranscriptomics with targeted metabolomics to resolve indole biosynthetic routes [177,190,191]. Stable isotope tracing of tryptophan can quantify flux from microbial pathways into circulating indoles and help partition host and microbial contributions [192,193,194]. Causal designs could include antibiotic perturbation with recovery, fecal microbiota transfer into gnotobiotic hosts, and longitudinal sampling across clinically relevant transitions [52,190,191]. Joint modeling of taxa, gene families, and indole derivatives will strengthen pathway attribution while maintaining analytic rigor [177,195,196].

Moving forward, the central challenge is to shift from descriptive associations toward mechanistic causation [131,197,198]. Stable-isotope-based in vivo flux tracing could clarify how KMO and KAT activities dynamically shape regional metabolite pools under physiological and stress conditions [197,199]. Complementary pharmacologic or genetic rescue experiments, targeting either KMO suppression or KAT reconstitution, will be critical to determine reversibility and compensatory limits of the pathway [128,131,197]. Behavioral paradigms that introduce stress load or learning demands should be layered on top of biochemical profiling to reveal context-dependent vulnerabilities [198,199,200,201,202].

Assays that tax prefrontal hippocampal control, including attentional set shifting, reversal learning, and contextual extinction, and assays that probe striatal gating under load, including progressive ratio and effort discounting, should be most sensitive to unmask domain-specific vulnerability [203,204,205]. Coupling these behaviors to region-resolved metabolomics and pathway indices, such as QA to KYNA and 3 HK to KYNA, plus AA, plus XA, will determine whether the cortical and hippocampal KYNA decrease with striatal KYNA increase marks resilience at baseline, yet reduces reserve under demand [139,206,207].

Spatial metabolomics, single-cell resolution analytics, and mesoscale circuit physiology offer the means to connect biochemical imbalances with cellular and network-level adaptations [197,199,202,208,209]. Finally, microbiome manipulation stands as a tractable lever to probe gut–brain indole inputs [131,197,199,210,211,212]. Integrating these platforms will close the loop from correlation to causation, refining translational targets across psychiatric and neurodegenerative disease contexts [202,213,214,215].

KAT II loss establishes a distinctive biochemical landscape marked by a KMO-driven tilt, reduced pterin support, and modulation through indole intermediates. This state disproportionately burdens affective and motor circuits, as evidenced by elevated 3-HK, reduced XA, and region-specific KYNA/QAA shifts, while sparing cognition and sociability under baseline conditions. Such dissociation between neurochemical disequilibria and behavioral resilience highlights the selective vulnerability of motor and emotional nodes. Viewed together, region-specific metabolic rewiring offers a parsimonious account of preserved baseline behavior through compensatory stabilization while flagging circuit and task-dependent vulnerability that is predicted to surface during cognitive challenge or stress. Crucially, these pathway imbalances converge into a coherent, biomarker-ready framework that not only clarifies the mechanistic underpinnings of Trp metabolism but also provides a tractable platform for targeted intervention strategies in neuropsychiatric disease contexts.

## 5. Conclusions

This work integrates region-resolved metabolomics across the STR, CTX, HIPP, CER, and STEM with enzyme-ratio proxies, cofactor mapping, and behavioral readouts. A convergent central signature emerges: 3-HK increases and XA decreases pan-regionally, KYNA reduction localizes to CTX and HIPP, KMO activity indices rise while KAT III flux falls, and oxidative-stress measures increase broadly, with a hippocampal-specific rise in the excitotoxicity index [33]. Cofactor analyses identify BIO depletion in CER and STEM and BH2 loss in CER. Gut–brain and serotonergic markers also shift, with hippocampal IAA reduction, cortical and cerebellar ICA elevation, and reduced cortical 5-HT turnover, yet cognition, sociability, and coordination remain intact. While these preserved functions suggest circuit-level compensation, the current behavioral battery was limited to baseline conditions. Stress paradigms or cognitively demanding tasks could potentially unmask subtle or latent deficits that remain silent under low-load conditions. Recognizing this limitation refines the interpretation of apparent resilience and underscores the value of incorporating such paradigms in future investigations to delineate compensatory versus genuinely preserved function. Consistent with region-specific rewiring rather than a uniform *kat2* effect, we note a paradoxical pattern: striatal KYNA elevation likely reflects compensatory KAT isoforms or astrocyte-mediated KYN shunting; forebrain bias toward AA aligns with oxidative-stress-driven KMO→KYNU flux that diverts 3-HK from KYNA. Taken together, the KAT II/*kat2* loss establishes a reproducible brain–periphery signature (KYNA↓, 3-HK↑, region-tuned KMO/KAT flux) without broad baseline behavioral deficits—best interpreted as evidence of circuit rewiring that buffers output, i.e., whole-axis metabolic disturbance does not immediately translate into behavior [216]. This framing motivates mechanism-targeted interventions (e.g., KMO inhibition, cofactor restoration, antioxidant support) and argues for biomarker-informed stratification [184]. Finally, because microbiome profiling was outside the present scope, ICA/IAA/INS findings should be viewed as hypothesis-generating for targeted microbiome-manipulation studies. In sum, findings delineate a KMO-tilted, pterin-constrained, indole-modulated milieu that selectively burdens affective and motor subdomains while sparing core cognitive and social functions.

## Figures and Tables

**Table 1:** Crosswalk of behavioral endpoints and regional metabolic indices. This provides a reader guide that aligns behavioral endpoints with region-specific metabolic indices using shared labels and panel order. Each row lists the task, the regions emphasized in the corresponding metabolite panels, and the indices displayed in figures and Supplementary Tables.

Domain	Behavioral Task	Primary Regions Referenced	Key Indices Listed	Figure Panels
Cognition	NORT	CTX, HIPP	KYNA, 3-HK, XA, QA/KYNA	Figure 2a; Figure 3b,e,f; Figure 5
Attention	OBAT	CTX, HIPP	KYNA, 3-HK, XA	Figure 2b; Figure 3b,e,f; Figure 5
Working memory	Y-maze	HIPP, CTX	KYNA, 3-HK, QA to KYNA	Figure 3b,e; Figure 5
Sociability	3CT sociability	CTX, CER	ICA, IAA, 5-HIAA	Figure 2c,d; Figure 4c,d; Figure 5
Social novelty	3CT novelty preference	CTX, CER, HIPP	ICA, IAA, KYNA	Figure 2c,d; Figure 4c,d; Figure 3b; Figure 5
Motor coordination	Rotarod	STR, CER	KYNA, 3-HK, AA, XA	Figure 3b,d–f; Figure 5
Affective proxy	MBT	HIPP, STEM	3-HK to KYNA plus AA plus XA	Figure 5; Figure 7a
Monoamine milieu	Task-agnostic pairing	CTX, STEM	BH4, BH2, BIO, DA cascade	Figure 4; Figure 5

AA, anthranilic acid; BIO, biopterin; BH2, dihydrobiopterin; BH4, tetrahydrobiopterin; CTX, cortex; DA, dopamine; HIPP, hippocampus; ICA, indole 3 carboxaldehyde; IAA, indole 3 acetic acid; 3-HK, 3 hydroxykynurenine; KYNA, kynurenic acid; QA, quinolinic acid; STR, striatum; CER, cerebellum; STEM, brainstem.

## Data Availability

The original contributions presented in this study are included in the article. Further inquiries can be directed to the corresponding authors.

## Supplementary Materials

The following supporting information can be downloaded at: https://www.mdpi.com/article/10.3390/cells14211711/s1. Supplement to the description of Section 2.3 Genotyping with TaqMan allelic discrimination assay in the Materials and Methods part of the manuscript; Table S1. Behavioral performance in WT and *kat2^−/−^* mice across multiple cognitive, social, and motor tests (NORT, OBAT, Y-Maze, MBT, 3CT, and Rotarod). Analyses revealed no significant inter-strain differences. Table S2. Comparative behavioral performance of wild-type (WT) and *kat2^−/−^* mice in object recognition (NORT, OBAT) and social interaction/novelty preference (3CT). Table S3. Regional brain concentrations of tryptophan (Trp) and its downstream metabolites in wild-type (WT) and *kat2^−/−^* mice across striatum, cortex, hippocampus, cerebellum, and brainstem. Table S4. Concentrations of indole-pyruvate and tyrosine-dopamine pathway metabolites in wild-type (WT) and *kat2^−/−^* mice in plasma and urine. Table S5. Ratios of kynurenine (KYN), serotonin (5-HT), indole–pyruvate, and tyrosine (Tyr)–dopamine (DA) metabolites to their precursors and associated enzyme activities across brain regions in wild-type (WT) and *kat2^−/−^* mice. Activities were estimated using product-to-substrate ratios for key enzymatic steps. Table S6. Ratios of oxidant/antioxidant and N-methyl-D-aspartate (NMDA) agonist/antagonist metabolites across brain regions in wild-type (WT) and *kat2^−/−^* mice. Regional indices of oxidative stress and excitotoxicity in WT and *kat2^−/−^* mice.

## Abbreviations

The following abbreviations are used in this manuscript:

3-HAA	3-hydroxyanthranilic acid
3-HK	3-hydroxykynurenine
3CT	three-chamber test
5-HT	5-hydroxytryptamine (serotonin)
5-HTP	5-hydroxytryptophan
5-HIAA	5-hydroxyindoleacetic acid
AA	anthranilic acid
AADC	aromatic L-amino acid decarboxylase
ALDH	aldehyde dehydrogenase
ARRIVE	animal research: reporting of in vivo experiments
ASD	autism spectrum disorder
BBB	blood–brain barrier
BH2	dihydrobiopterin
BH4	tetrahydrobiopterin
BIO	biopterin
CER	cerebellum
CNS	central nervous system
COMT	catechol-O-methyltransferase
CTX	cortex
DA	dopamine
DI	discrimination index
DOPAC	3,4-dihydroxyphenylacetic acid
GAD	generalized anxiety disorder
HIPP	hippocampus
HVA	homovanillic acid
IAA	indole-3-acetic acid
ICA	indole-3-carboxylic acid
INS	indoxyl sulfate
KAT	kynurenine aminotransferase
KMO	kynurenine 3-monooxygenase
KYN	kynurenine
KYNA	kynurenic acid
KYNU	kynureninase
L-DOPA	dihydroxyphenylalanine/levodopa
LC-MS	liquid chromatography–tandem mass spectrometry
MAO	monoamine oxidase
MBT	marble-burying test
MDD	major depressive disorder
MEL	melatonin
MHPGS	3-methoxy-4-hydroxyphenylglycol sulfate
NMDA	N-methyl-D-aspartate
NORT	novel object recognition test
OBAT	object-based attention test
PI	preference index
QA	quinolinic acid
QAA	quinaldic acid
SCZ	schizophrenia
STEM	brainstem
STR	striatum
Trp	tryptophan
TPH	tryptophan hydroxylase
Tyr	tyrosine
UHPLC-MS	ultra-high-performance liquid chromatography–tandem mass spectrometry
VMA	vanillylmandelic acid
WT	wild-type
XA	xanthurenic acid
