# Supplementary material for: Behavioral Balance in Tryptophan Turmoil: Regional Metabolic Rewiring in Kynurenine Aminotransferase II Knockout Mice
Source: Cells. 2025 Oct 31;14(21):1711. doi: 10.3390/cells14211711 (PMC12607557; doi:10.3390/cells14211711)
Supplement: Supplementary file 1 [file cells-14-01711-s001.zip › cells-3908036-supplementary.pdf]

cells

MDPI

Supplement to the description of section 2.3 Genotyping with TaqMan allelic discrimination assay in the Materials and Methods part of the manuscript.

All animals were genotyped in advance, ensuring verification before the subsequent experimental studies. Mice were anesthetized with 2% isoflurane, and following local analgesia with 5% lidocaine ointment, a 3 mm fragment of the tail was excised using sterile instruments under aseptic conditions. Tissue samples were stored at -80°C until further processing. For DNA extraction, 75 µl of a freshly prepared lysis buffer containing equal volumes of 25 mM NaOH and 0.2 mM disodium EDTA was added to each sample. After incubation at 95°C for 30 minutes, the suspension was cooled to 4°C and then neutralized with 75 µl of 40 mM TRIS-HCl buffer. This method was adapted from the HotSHOT protocol and consistently yields DNA suitable for reliable genotyping, as previously demonstrated by Truett et al. DNA concentration and purity were assessed using a NanoDrop spectrophotometer (MaestroGen, Taipei, Taiwan). The resulting DNA extracts were stored at -20°C until analysis.

Genotyping was performed using a fluorescence-labeled TaqMan allelic discrimination assay. The forward primer sequence was 5′–TAACAGTGCATCCCGAGTGA–3′, the reverse primer sequence was 5′–GAGGGCTCTGGCTTTGTTTT–3′, while probe 1 and probe 2 sequences were 5′–6-FAM-CAACGAGCCTGGCCAGAA-BHQ-1–3′ and 5′–HEX-TGCAACGACTGGCCAGAAAG-BHQ-1–3′, respectively (Metabion, Steinkirchen / Planegg, Germany). For each reaction, the PCR was assembled using the following reagents: PCR Master Mix (5 µl; PCRBiosystems, London, UK), forward primer (1 µl), reverse primer (1 µl), probe 1 (0.5 µl), probe 2 (0.5 µl), DNA template (1 µl), and water (1 µl). Non-template control reactions contained water instead of DNA. Reaction mixtures were aliquoted into 96-well plates. PCR amplification and allelic discrimination were performed in single-plex reactions using a CFX Opus 96 Real-Time PCR System (Bio-Rad Laboratories, Hercules, California, USA) according to the manufacturer's instructions. The amplification protocol consisted of an initial denaturation at 95°C for 10 min, followed by 40 cycles of 92°C for 15 sec and 60°C for 1 min. Fluorescence data were analyzed with CFX Maestro software.

This approach ensured that all mutant animals included in the experiments were confirmed to carry the targeted genetic modification, and wild-type mice were correctly identified as controls.

Table S1. Behavioral performance in WT and *kat2*$^{-/-}$ mice across multiple cognitive, social, and motor tests (NORT, OBAT, Y-Maze, MBT, 3CT, and Rotarod). Analyses revealed no significant inter-strain differences.

Behavioral Test Type	Number of Animals (WT/*kat2*$^{-/-}$)	Phase of the Test	Parameter of the Test	WT Mean ± SD	*kat2*$^{-/-}$ Mean ± SD	*p*-Value
NORT	12/12	Testing phase	Time spent with familiar object (s)	17.500 ± 8.635	18.556 ± 11.886	$p < 0.839$
			Time spent with novel object (s)	66.250 ± 59.461	72.444 ± 38.730	$p < 0.596$
			Discrimination index	0.455 ± 0.321	0.592 ± 0.141	$p < 0.294$
			Preference	72.760	79.604	$p < 0.293$

			index	± 16.032	± 7.061	
OBAT	12/12	Testing phase	Time spent with familiar object (s)	15.362 ± 7.437	10.729 ± 6.786	$p < 0.905$
			Time spent with novel object (s)	19.747 ± 7.820	15.796 ± 6.372	$p < 0.268$
			Discrimination index	0.136 ± 0.239	0.239 ± 0.287	$p < 0.428$
			Preference index (%)	56.802 ± 11.943	61.944 ± 14.355	$p < 0.428$
Y-maze	12/12	-	Spontaneous alternations (%)	52.833 ± 27.996	66.500 ± 18.880	$p < 0.175$
			Number of total entries	15.583 ± 11.579	18.000 ± 15.788	$p < 0.954$
MBT	10/13	-	Buried marbles	5.467 ± 4.207	6.133 ± 4.121	$p < 0.738$
			Partially buried marbles	4.267 ± 2.344	4.533 ± 2.326	$p < 0.757$
			Displaced marbles	1.733 ± 1.870	1.333 ± 1.345	$p < 0.731$
			Intact marbles	4.533 ± 3.248	4.000 ± 2.976	$p < 0.643$
3CT	12/12	Testing sociability	Time in social chamber (s)	265.717 ± 40.368	260.658 ± 54.993	$p < 0.799$
			Time in non-social chamber (s)	247.748 ± 25.751	247.988 ± 56.056	$p < 0.989$
			Time in center chamber (s)	86.536 ± 32.148	91.355 ± 28.005	$p < 0.699$
			Sniffing social cage (s)	145.955 ± 39.690	136.336 ± 37.149	$p < 0.546$
			Sniffing non-social cage (s)	114.603 ± 33.637	117.447 ± 33.452	$p < 0.837$
			Total sniffing time (s)	260.558 ± 38.784	253.783 ± 47.129	$p < 0.704$
			Social chamber entries (number)	12.667 ± 3.725	13.417 ± 4.621	$p < 0.666$
			Non-social chamber entries (number)	13.167 ± 4.174	12.833 ± 4.687	$p < 0.855$
			Total entries (number)	25.833 ± 7.673	26.250 ± 9.245	$p < 0.905$
		Testing novelty preference	Time in novel chamber (s)	263.188 ± 60.124	253.058 ± 68.641	$p < 0.704$
			Time in familiar chamber (s)	238.687 ± 55.961	237.654 ± 56.502	$p < 0.964$
			Time in center chamber (s)	98.126 ± 40.008	109.288 ± 53.470	$p < 0.568$
			Sniffing novel animal's cage (s)	129.261 ± 50.164	109.373 ± 44.085	$p < 0.313$
			Sniffing familiar animal's cage (s)	95.015 ± 51.306	92.903 ± 62.090	$p < 0.928$
			Total sniffing time (s)	224.276 ± 75.342	202.276 ± 79.270	$p < 0.493$

			Novel chamber entries (number)	9.917 ± 3.450	11.083 ± 3.679	$p < 0.431$
			Familiar chamber entries (number)	10.250 ± 3.279	11.167 ± 4.764	$p < 0.589$
			Total entries (number)	20.167 ± 6.548	22.250 ± 7.979	$p < 0.492$
Rotarod	12/12	-	Mean time spent on the rod	100.428 ± 35.017	89.708 ± 41.453	$p < 0.501$

Mean ± SD. 3CT, three-chamber test; MBT, marble burying test; NORT, novel object recognition test; OBAT, object-based attention test.

Table S2. Comparative behavioral performance of wild-type (WT) and *kat2*$^{-/-}$ mice in object recognition (NORT, OBAT) and social interaction/novelty preference (3CT). In the NORT, both WT and *kat2*$^{-/-}$ mice demonstrated a significant preference for the novel object. In the OBAT, the mutant strain exhibited greater exploration of the novel object. In the 3CT, both genotypes spent more time in the side chambers than in the center chamber during both the sociability and novelty preference phases.

**Test Type**	**Phase of the Test**	**WT**			***kat2*$^{-/-}$**		
NORT	Testing phase	**Sniffing familiar object (s)**	**Sniffing novel object (s)**	***p*-value**	**Sniffing familiar object (s)**	**Sniffing novel object (s)**	***p*-value**
		17.500 ± 8.635	66.250 ± 59.461	$p < 0.018$ *	18.556 ± 11.886	72.444 ± 38.730	$p < 0.001$ ***
OBAT	Testing phase	**Sniffing familiar object (s)**	**Sniffing novel object (s)**		**Sniffing familiar object (s)**	**Sniffing novel object (s)**	
		15.362 ± 7.437	19.747 ± 7.820	$p < 0.081$	10.729 ± 6.786	15.796 ± 6.372	$p < 0.039$ *
3CT	Testing sociability	**Time in social chamber (s)**	**Time in center chamber (s)**		**Time in social chamber (s)**	**Time in center chamber (s)**	
		265.717 ± 40.368	86.536 ± 32.148	$p < 0.001$ ***	260.658 ± 54.993	91.355 ± 28.005	$p < 0.001$ ***
		**Time in non-social chamber (s)**	**Time in center chamber (s)**		**Time in non-social chamber (s)**	**Time in center chamber (s)**	
		247.748 ± 25.751	86.536 ± 32.148	$p < 0.001$ ***	247.988 ± 56.056	91.355 ± 28.005	$p < 0.001$ ***
		**Time in social chamber (s)**	**Time in non-social chamber (s)**		**Time in social chamber (s)**	**Time in non-social chamber (s)**	
		265.717 ± 40.368	247.748 ± 25.751	$p < 0.319$	260.658 ± 54.993	247.988 ± 56.056	$p < 0.691$
		**Sniffing social cage (s)**	**Sniffing non-social cage (s)**		**Sniffing social cage (s)**	**Sniffing non-social cage (s)**	
		145.955 ± 39.690	114.603 ± 33.637	$p < 0.110$	136.336 ± 37.149	117.447 ± 33.452	$p < 0.240$
		**Social chamber entries**	**Non-social chamber entries**		**Social chamber entries**	**Non-social chamber entries**	

	**(number)**	**(number)**		**(number)**	**(number)**	
	12.667 ± 3.725	13.167 ± 4.174	$p < 0.389$	13.417 ± 4.621	12.833 ± 4.687	$p < 0.089$
Testing novelty preference	**Time in novel chamber (s)**	**Time in center chamber (s)**		**Time in novel chamber (s)**	**Time in center chamber (s)**	
	263.188 ± 60.124	98.126 ± 40.008	$p < 0.001$ ***	253.058 ± 68.641	109.288 ± 53.470	$p < 0.002$ **
	**Time in familiar chamber (s)**	**Time in center chamber (s)**		**Time in familiar chamber (s)**	**Time in center chamber (s)**	
	238.687 ± 55.961	98.126 ± 40.008	$p < 0.001$ ***	237.654 ± 56.502	109.288 ± 53.470	$p < 0.001$ ***
	**Time in novel chamber (s)**	**Time in familiar chamber (s)**		**Time in novel chamber (s)**	**Time in familiar chamber (s)**	
	263.188 ± 60.124	238.687 ± 55.961	$p < 0.453$	253.058 ± 68.641	237.654 ± 56.502	$p < 0.754$
	**Sniffing novel animal's cage (s)**	**Sniffing familiar animal's cage (s)**		**Sniffing novel animal's cage (s)**	**Sniffing familiar animal's cage (s)**	
	129.261 ± 50.164	95.015 ± 51.306	$p < 0.109$	109.373 ± 44.085	92.903 ± 62.090	$p < 0.530$
	**Novel chamber entries (number)**	**Familiar chamber entries (number)**		**Novel chamber entries (number)**	**Familiar chamber entries (number)**	
	9.917 ± 3.450	10.250 ± 3.279	$p < 0.474$	11.083 ± 3.679	11.167 ± 4.764	$p < 0.681$

Statistical significance was assessed for within-group contrasts (familiar vs. novel; social vs. non-social; chamber comparisons), and p-values are reported. Mean ± SD. Asterisks denote significance levels: *, $p < 0.05$, **, $p < 0.01$, ***, $p < 0.001$. 3CT, three-chamber test; *kat2*$^{-/-}$, kynurenine aminotransferase II knockout mice; NORT, novel object recognition test; OBAT, object-based attention test; WT, wild-type mice.

Table S3. Regional brain concentrations of tryptophan (Trp) and its downstream metabolites in wild-type (WT) and *kat2*$^{-/-}$ mice across striatum, cortex, hippocampus, cerebellum, and brainstem.

Metabolite	Striatum (nM)			Cortex (nM)			Hippocampus (nM)			Cerebellum (nM)			Brainstem (nM)		
	Mean ± SD		*p*-Value	Mean ± SD		*p*-Value	Mean ± SD		*p*-Value	Mean ± SD		*p*-Value	Mean ± SD		*p*-Value
	WT	*kat2*$^{-/-}$		WT	*kat2*$^{-/-}$		WT	*kat2*$^{-/-}$		WT	*kat2*$^{-/-}$		WT	*kat2*$^{-/-}$	
Trp	25511.111 ± 5688.243	21988.889 ± 1940.647	$p < 0.098$	30780.000 ± 5921.674	24090.000 ± 2431.026	$p < 0.004$ **	30433.333 ± 7230.664	25000.000 ± 2256.103	$p < 0.047$ *	29530.000 ± 6997.468	25410.000 ± 2154.814	$p < 0.092$	29210.000 ± 8273.579	24730.000 ± 2685.786	$p < 0.121$
KYN	146.444 ± 32.423	152.000 ± 45.031	$p < 0.768$	123.880 ± 27.763	121.230 ± 40.829	$p < 0.867$	126.822 ± 25.699	124.600 ± 38.033	$p < 0.886$	204.000 ± 173.369	129.880 ± 56.356	$p < 0.215$	132.130 ± 37.311	119.560 ± 41.100	$p < 0.483$
KYNA	1.757 ± 0.623	3.561 ± 2.124	$p < 0.026$ *	5.428 ± 3.032	2.963 ± 1.108	$p < 0.027$ *	2.683 ± 0.885	1.787 ± 0.647	$p < 0.026$ *	5.347 ± 0.925	4.832 ± 1.728	$p < 0.417$	4.315 ± 1.303	3.273 ± 0.974	$p < 0.058$
QAA	no data	no data	no data	0.812 ± 0.298	0.714 ± 0.127	$p < 0.349$	0.765 ± 0.216	0.807 ± 0.188	$p < 0.671$	0.416 ± 0.210	0.249 ± 0.113	$p < 0.040$ *	0.469 ± 0.323	0.171 ± 0.102	$p < 0.012$ *
AA	2.534 ± 0.771	2.431 ± 1.145	$p < 0.825$	0.320 ± 0.089	0.461 ± 0.176	$p < 0.036$ *	0.323 ± 0.136	0.213 ± 0.077	$p < 0.050$	0.400 ± 0.148	0.296 ± 0.193	$p < 0.192$	0.554 ± 0.209	0.477 ± 0.308	$p < 0.523$
3-HK	43.078 ± 7.418	78.644 ± 40.163	$p < 0.019$ *	50.230 ± 15.967	87.210 ± 42.381	$p < 0.019$ *	57.667 ± 15.585	104.467 ± 41.026	$p < 0.006$ **	77.460 ± 30.378	118.100 ± 51.104	$p < 0.044$ *	47.070 ± 12.161	83.740 ± 35.505	$p < 0.006$ **
XA	1.906 ± 1.161	1.342 ± 0.662	$p < 0.224$	7.316 ± 5.644	2.516 ± 1.631	$p < 0.019$ *	1.698 ± 0.800	0.885 ± 0.406	$p < 0.015$ *	2.402 ± 0.838	1.349 ± 0.903	$p < 0.015$ *	3.945 ± 2.037	1.324 ± 0.854	$p < 0.001$ ***
3-HAA	no data	no data	no data	6.581 ± 2.409	10.128 ± 3.732	$p < 0.021$ *	3.820 ± 1.574	3.994 ± 2.532	$p < 0.863$	no data	no data	no data	no data	no data	no data
QA	20.337 ± 11.449	32.537 ± 15.421	$p < 0.075$	35.650 ± 11.656	32.946 ± 20.266	$p < 0.719$	18.296 ± 7.872	24.667 ± 11.260	$p < 0.183$	27.057 ± 21.057	20.015 ± 12.182	$p < 0.372$	28.390 ± 16.309	30.201 ± 13.870	$p < 0.792$
PA	223.877 ± 45.544	233.296 ± 67.819	$p < 0.734$	146.707 ± 43.541	171.643 ± 46.369	$p < 0.231$	165.885 ± 81.710	146.251 ± 46.419	$p < 0.540$	213.970 ± 57.567	291.345 ± 135.202	$p < 0.152$	220.273 ± 63.283	246.122 ± 89.874	$p < 0.467$
**Serotonin pathway**															
5-HTP	55.689 ± 11.051	37.989 ± 20.492	$p < 0.037$ *	80.070 ± 26.609	37.490 ± 15.897	$p < 0.001$ ***	48.167 ± 15.514	42.011 ± 9.346	$p < 0.323$	11.708 ± 4.638	6.451 ± 2.871	$p < 0.007$ **	65.120 ± 19.882	65.200 ± 34.451	$p < 0.995$
5-HT	2854.444 ± 281.741	3087.778 ± 318.856	$p < 0.119$	2780.000 ± 364.722	3238.000 ± 304.478	$p < 0.007$ **	3162.222 ± 368.571	3316.667 ± 482.519	$p < 0.457$	382.600 ± 182.449	423.500 ± 218.472	$p < 0.655$	3542.000 ± 426.375	3554.000 ± 334.006	$p < 0.945$
5-HIAA	2771.111 ± 232.886	2825.556 ± 493.739	$p < 0.769$	2816.000 ± 511.147	2634.000 ± 333.340	$p < 0.358$	3984.444 ± 640.666	3618.889 ± 412.566	$p < 0.169$	952.300 ± 165.692	954.100 ± 216.632	$p < 0.984$	3998.000 ± 733.664	4076.000 ± 651.804	$p < 0.804$
**Indole-pyruvate pathway**															

IAA	263.000 ± 94.166	196.111 ± 59.711	$p < 0.091$	174.300 ± 46.294	160.100 ± 32.385	$p < 0.437$	179.000 ± 41.985	113.767 ± 30.709	$p < 0.002$ **	47.260 ± 26.084	62.300 ± 18.091	$p < 0.151$	126.790 ± 38.684	106.450 ± 17.163	$p < 0.146$
ICA	52.522 ± 15.104	67.000 ± 25.999	$p < 0.168$	53.590 ± 10.768	85.520 ± 23.399	$p < 0.001$ ***	58.078 ± 12.274	48.344 ± 7.459	$p < 0.059$	46.850 ± 16.225	70.060 ± 31.928	$p < 0.055$	57.430 ± 18.870	56.190 ± 21.972	$p < 0.894$
IPA	no data	no data	no data	no data	no data	no data	no data	no data	no data	14.509 ± 7.547	12.995 ± 6.244	$p < 0.631$	29.370 ± 14.081	19.479 ± 5.821	$p < 0.055$
ILA	88.656 ± 49.392	54.078 ± 17.784	$p < 0.066$	91.820 ± 24.495	74.690 ± 11.764	$p < 0.062$	122.067 ± 43.164	91.444 ± 23.630	$p < 0.080$	62.750 ± 18.591	59.500 ± 12.143	$p < 0.649$	106.910 ± 19.352	68.030 ± 12.774	$p < 0.001$ ***
INS	136.444 ± 124.642	66.144 ± 22.154	$p < 0.115$	181.320 ± 108.171	129.140 ± 54.474	$p < 0.190$	102.700 ± 93.846	48.411 ± 15.970	$p < 0.106$	114.680 ± 46.885	73.380 ± 32.000	$p < 0.034$ *	135.570 ± 66.490	86.080 ± 27.670	$p < 0.043$ *
pCS	22.863 ± 45.643	6.428 ± 3.057	$p < 0.297$	13.201 ± 15.641	6.452 ± 6.191	$p < 0.221$	11.421 ± 14.434	2.932 ± 1.870	$p < 0.099$	28.415 ± 45.171	4.284 ± 2.878	$p < 0.109$	5.987 ± 5.293	4.280 ± 4.371	$p < 0.442$
**Tyrosine-dopamine pathway**															
Tyr	55533.333 ± 25146.620	47200.000 ± 66.11.354	$p < 0.351$	74760.000 ± 27036.856	55710.000 ± 9047.216	$p < 0.049$ *	76522.222 ± 33835.513	57944.444 ± 10032.337	$p < 0.134$	72320.000 ± 27354.983	57480.000 ± 6273.542	$p < 0.112$	67800.000 ± 25030.026	53600.000 ± 9267.026	$p < 0.110$
L-DOPA	no data	no data	no data	130.840 ± 71.182	119.180 ± 91.862	$p < 0.755$	147.389 ± 60.587	122.400 ± 31.455	$p < 0.288$	97.580 ± 16.040	103.530 ± 54.909	$p < 0.746$	144.090 ± 139.318	118.740 ± 19.909	$p < 0.576$
3OMD	43.067 ± 11.017	48.467 ± 22.082	$p < 0.521$	42.010 ± 7.379	46.620 ± 10.305	$p < 0.265$	42.867 ± 13.189	41.678 ± 9.240	$p < 0.828$	44.220 ± 8.036	38.350 ± 6.488	$p < 0.089$	36.990 ± 11.834	35.540 ± 8.342	$p < 0.755$
DA	238805.321 ± 62124.925	226596.946 ± 85742.994	$p < 0.734$	11460.000 ± 4415.938	9733.000 ± 1997.838	$p < 0.265$	327.778 ± 184.660	301.556 ± 119.914	$p < 0.726$	126.260 ± 129.803	90.500 ± 32.822	$p < 0.409$	373.800 ± 137.416	320.700 ± 78.006	$p < 0.302$
3-MT	12277.778 ± 3429.001	15417.778 ± 7109.386	$p < 0.250$	2637.000 ± 1213.370	3510.000 ± 2184.272	$p < 0.284$	72.811 ± 27.012	76.333 ± 42.626	$p < 0.837$	48.360 ± 51.290	39.780 ± 27.131	$p < 0.646$	108.990 ± 55.943	100.420 ± 45.985	$p < 0.713$
DOPAC	9731.111 ± 2098.681	8508.889 ± 2173.571	$p < 0.243$	3231.000 ± 662.528	2604.000 ± 1298.026	$p < 0.190$	99.844 ± 31.080	119.122 ± 46.575	$p < 0.317$	118.510 ± 70.272	58.230 ± 22.991	$p < 0.019$ *	286.600 ± 83.187	269.700 ± 90.096	$p < 0.668$
HVA	8845.556 ± 1621.983	9957.778 ± 2933.235	$p < 0.334$	3219.000 ± 465.271	2994.000 ± 815.383	$p < 0.458$	563.667 ± 249.747	622.000 ± 272.426	$p < 0.642$	241.000 ± 72.399	235.410 ± 110.270	$p < 0.895$	512.900 ± 142.529	479.100 ± 199.441	$p < 0.668$
VMA	no data	no data	no data	6.815 ± 4.531	9.876 ± 7.149	$p < 0.268$	4.113 ± 2.537	7.131 ± 3.580	$p < 0.056$	23.772 ± 34.420	34.862 ± 38.254	$p < 0.504$	7.121 ± 4.828	8.801 ± 6.926	$p < 0.537$
MHPGS	31.467 ± 15.193	33.022 ± 10.663	$p < 0.805$	42.420 ± 20.320	47.070 ± 7.532	$p < 0.506$	49.722 ± 16.118	46.011 ± 9.992	$p < 0.565$	23.668 ± 27.307	17.066 ± 6.494	$p < 0.467$	21.700 ± 9.295	22.040 ± 5.288	$p < 0.921$
BIO	43.611 ± 16.815	55.533 ± 18.984	$p < 0.178$	22.410 ± 7.193	18.823 ± 10.159	$p < 0.374$	15.201 ± 11.095	14.789 ± 12.229	$p < 0.941$	15.690 ± 3.897	9.233 ± 3.056	$p < 0.001$ ***	62.380 ± 19.428	41.020 ± 9.643	$p < 0.006$ **
BH2	514.956 ± 164.623	441.378 ± 173.194	$p < 0.369$	no data	no data	no data	no data	no data	no data	117.947 ± 16.544	76.528 ± 22.014	$p < 0.001$ ***	222.409 ± 82.207	192.147 ± 59.659	$p < 0.359$

Mean ± SD. Asterisks indicate significance levels: *, $p < 0.05$; **, $p < 0.01$; ***, $p < 0.001$. 3-HAA, 3-hydroxyanthranilic acid; 3-HK, 3-hydroxykynurenine; 3-MT, 3-methoxytyramine; 5-HIAA, 5-hydroxyindoleacetic acid; 5-HT, serotonin (5-hydroxytryptamine); 5-HTP, 5-hydroxytryptophan; AA, anthranilic acid; BH2, dihydrobiopterin; BIO, biopterin; DA, dopamine; DOPAC, 3,4-dihydroxyphenylacetic acid; HVA, homovanillic acid; ICA, indole-3-carboxaldehyde; IAA, indole-3-acetic acid; ILA, indole-3-lactic acid; INS, indoxyl sulfate; IPA, indole-3-propionic acid; KYN, kynurenine; KYNA, kynurenic acid; L-DOPA, levodopa; 3OMD, 3-O-methyldopa; MHPGS, 3-methoxy-4-hydroxyphenylglycol sulfate; PA, picolinic acid; pCS, p-Cresyl sulfate; QA, quinolinic acid; QAA, quinaldic acid; Trp, tryptophan; Tyr, tyrosine; VMA, vanillylmandelic acid; XA, xanthurenic acid.

Table S4. Concentrations of indole-pyruvate and tyrosine-dopamine pathway metabolites in wild-type (WT) and $kat2^{-/-}$ mice in plasm and urine.

Metabolite	Plasm (nM)			Urine (nM)		
	Mean ± SD		*p*-Value	Mean ± SD		*p*-Value
	WT	$kat2^{-/-}$		WT	$kat2^{-/-}$	
**Indole-pyruvate pathway**						
ICA	no data	no data	no data	no data	no data	no data
IPA	no data	no data	no data	no data	no data	no data
ILA	no data	no data	no data	no data	no data	no data
pCS	853.520 ± 961.663	1097.193 ± 1196.572	$p < 0.622$	9683.873 ± 15558.939	7429.639 ± 12598.662	$p < 0.726$
**Tyrosine-dopamine pathway**						
Tyr	50824.432 ± 20811.617	35775.857 ±16975.863	$p < 0.093$	9411.420 ± 2214.266	8789.288 ± 1547.575	$p < 0.476$
L-DOPA	36.800 ± 15.606	35.109 ± 13.708	$p < 0.800$	no data	no data	no data
3OMD	36.340 ± 5.556	31.128 ± 5.595	$p < 0.051$	41.828 ± 21.255	40.299 ± 17.829	$p < 0.864$
DA	no data	no data	no data	671.105 ± 320.951	779.887 ± 193.877	$p < 0.371$
3-MT	2.653 ± 1.315	1.796 ± 0.655	$p < 0.082$	241.517 ± 93.336	235.939 ± 50.578	$p < 0.870$
DOPAC	no data	no data	no data	370.196 ± 224.797	301.471 ± 108.291	$p < 0.395$
HVA	no data	no data	no data	1120.520 ± 890.606	731.657 ± 173.621	$p < 0.192$
VMA	no data	no data	no data	820.064 ± 567.571	760.459 ± 124.132	$p < 0.749$
MHPGS	27.657 ± 12.496	15.392 ± 6.886	$p < 0.014$ *	8533.153 ± 3929.104	14639.178 ± 3364.617	$p < 0.002$ **
BIO	68.419 ± 23.013	82.535 ± 17.725	$p < 0.142$	231.328 ± 80.142	221.133 ± 88.018	$p < 0.790$
BH2	588.898 ± 122.352	577.701 ± 178.965	$p < 0.872$	4508.851 ± 2655.882	4488.304 ± 2298.353	$p < 0.985$

Mean ± SD. Asterisks indicate significance levels: *, $p < 0.05$; **, $p < 0.01$. 3OMD, 3-O-methyldopa; 3-MT, 3-methoxytyramine; BH2, dihydroxybiopterin; BIO, biopterin; DA, dopamine; DOPAC, 3,4-dihydroxyphenylacetic acid; HVA, homovanillic acid; ICA, indole-3-carboxaldehyde; ILA, indole-3-lactic acid; IPA, 3-indolepropionic acid; $kat2^{-/-}$, kynurenine aminotransferase II knockout mice; L-DOPA, levodopa; MHPGS, 3-methoxy-4-hydroxyphenylglycol sulphate; pCS, para-Cresol sulphate; SD, standard deviance; Tyr, tyrosine; VMA, vanillylmandelic acid; WT, wild-type mice.

Table S5. Ratios of kynurenine (KYN), serotonin (5-HT), indole-pyruvate and tyrosine (Tyr)-dopamine (DA) metabolites to their precursors and associated enzyme activities across brain regions in wild-type (WT) and *kat2*$^{-/-}$ mice. Activities were estimated using product-to-substrate ratios for key enzymatic steps.

Enzyme	Product/Substrate	Striatum			Cortex			Hippocampus			Cerebellum			Brainstem		
		Mean ± SD		p-Value	Mean ± SD		p-Value	Mean ± SD		p-Value	Mean ± SD		p-Value	Mean ± SD		p-Value
		WT	*kat2*$^{-/-}$		WT	*kat2*$^{-/-}$		WT	*kat2*$^{-/-}$		WT	*kat2*$^{-/-}$		WT	*kat2*$^{-/-}$	
TDO/IDOs	KYN/Trp	0.006 ± 0.001	0.007 ± 0.002	$p < 0.188$	0,004 ± 0,001	0.005 ± 0.001	$p < 0.212$	0.004 ± 0.001	0.005 ± 0.001	$p < 0.245$	0.006 ± 0.004	0.005 ± 0.002	$p < 0.349$	0.005 ± 0.001	0.005 ± 0.002	$p < 0.907$
KATs	KYNA/KYN	0.012 ± 0.005	0.026 ± 0.017	$p < 0.035$ *	0.045 ± 0.027	0.026 ± 0.012	$p < 0.063$	0.022 ± 0.010	0.015 ± 0.007	$p < 0.099$	0.036 ± 0.015	0.041 ± 0.019	$p < 0.487$	0.034 ± 0.011	0.032 ± 0.017	$p < 0.696$
KMO	3-HK/KYN	0.305 ± 0.075	0.510 ± 0.216	$p < 0.002$ **	0.413 ± 0.131	0.714 ± 0.262	$p < 0.001$ **	0.463 ± 0.121	0.844 ± 0.264	$p < 0.001$ ***	0.458 ± 0.139	0.957 ± 0.365	$p < 0.001$ ***	0.365 ± 0.087	0.709 ± 0.230	$p < 0.001$ ***
KYNU	AA/KYN	0.018 ± 0.006	0.018 ± 0.009	$p < 0.951$	0.003 ± 0.001	0.004 ± 0.003	$p < 0.208$	0.003 ± 0.001	0.002 ± 0.001	$p < 0.301$	0.003 ± 0.001	0.003 ± 0.002	$p < 0.913$	0.004 ± 0.002	0.005 ± 0.004	$p < 0.651$
KYNU	3-HAA/3-HK	no data	no data	no data	0.135 ± 0.040	0.125 ± 0.034	$p < 0.540$	0.072 ± 0.036	0.038 ± 0.020	$p < 0.026$ *	no data	no data	no data	no data	no data	no data
KAT III	XA/3-HK	0.045 ± 0.027	0.022 ± 0.018	$p < 0.052$	0.151 ± 0.106	0.035 ± 0.027	$p < 0.001$ **	0.031 ± 0.017	0.011 ± 0.009	$p < 0.007$ **	0.034 ± 0.015	0.014 ± 0.015	$p < 0.001$ **	0.088 ± 0.054	0.020 ± 0.017	$p < 0.001$ ***
3-HAO	QA/3-HAA	no data	no data	no data	5.910 ± 2.878	3.693 ± 2.022	$p < 0.112$	6.125 ± 4.070	10.220 ± 9.885	$p < 0.508$	no data	no data	no data	no data	no data	no data
3-HAO + ACMSD	PA/3-HAA	no data	no data	no data	23.688 ± 7.982	20.024 ± 13.316	$p < 0.082$	66.477 ± 82.924	51.852 ± 33.947	$p < 0.895$	no data	no data	no data	no data	no data	no data
TPHs	5-HTP/Trp	0.002 ± 0.000	0.002 ± 0.001	$p < 0.313$	0.003 ± 0.001	0.002 ± 0.001	$p < 0.005$ **	0.002 ± 0.000	0.002 ± 0.000	$p < 0.331$	0.000 ± 0.000	0.000 ± 0.000	$p < 0.003$ **	0.002 ± 0.001	0.003 ± 0.001	$p < 1.000$
AADC	5-HT/5-HTP	53.246 ± 12.810	110.941 ± 73.047	$p < 0.015$ *	37.833 ± 12.360	102.426 ± 43.532	$p < 0.001$ **	72.470 ± 26.437	82.603 ± 22.339	$p < 0.393$	34.597 ± 17.138	74.676 ± 41.567	$p < 0.004$ **	63.557 ± 38.662	68.894 ± 37.313	$p < 0.496$
MAOs + ALDH	5-HIAA/5-HT	0.982 ± 0.148	0.922 ± 0.180	$p < 0.453$	1.036 ± 0.280	0.822 ± 0.141	$p < 0.044$ *	1.290 ± 0.327	1.126 ± 0.285	$p < 0.274$	2.905 ± 1.239	2.539 ± 0.796	$p < 0.442$	1.150 ± 0.271	1.160 ± 0.232	$p < 0.926$
TMO (TrD, ArAT)	IAA/Trp	0.010 ± 0.003	0.009 ± 0.002	$p < 0.212$	0.006 ± 0.002	0.007 ± 0.001	$p < 0.054$	0.006 ± 0.001	0.005 ± 0.001	$p < 0.021$ *	0.002 ± 0.001	0.002 ± 0.001	$p < 0.026$ *	0.004 ± 0.001	0.004 ± 0.001	$p < 0.597$
TNA	INS/Trp	0.005 ± 0.003	0.003 ± 0.001	$p < 0.554$	0.006 ± 0.003	0.005 ± 0.002	$p < 0.624$	0.003 ± 0.002	0.002 ± 0.001	$p < 0.161$	0.004 ± 0.002	0.003 ± 0.001	$p < 0.170$	0.005 ± 0.003	0.003 ± 0.001	$p < 0.147$
TH	L-DOPA/Tyr	no data	no data	no data	0.002 ± 0.001	0.002 ± 0.002	$p < 0.968$	0.002 ± 0.001	0.002 ± 0.001	$p < 0.555$	0.001 ± 0.000	0.002 ± 0.001	$p < 0.126$	0.002 ± 0.001	0.002 ± 0.001	$p < 0.132$

AADC	DA/L-DOPA	no data	no data	no data	110.049 ± 70.406	132.849 ± 107.738	$p < 0.880$	2.583 ± 1.810	2.556 ± 1.048	$p < 0.969$	1.370 ± 1.513	1.101 ± 0.682	$p < 0.821$	3.589 ± 1.777	2.775 ± 0.933	$p < 0.216$
MAOs	DOPAC/DA	0.044 ± 0.020	0.043 ± 0.020	$p < 0.452$	0.320 ± 0.142	0.274 ± 0.140	$p < 0.290$	0.376 ± 0.178	0.427 ± 0.156	$p < 0.532$	1.324 ± 0.697	0.669 ± 0.210	$p < 0.023$ *	0.810 ± 0.238	0.909 ± 0.393	$p < 0.500$
COMT	HVA/DOPAC	0.944 ± 0.256	1.279 ± 0.584	$p < 0.143$	1.045 ± 0.290	1.444 ± 0.799	$p < 0.165$	6.137 ± 3.971	5.665 ± 2.558	$p < 0.895$	2.271 ± 0.755	4.685 ± 2.395	$p < 0.012$ *	1.933 ± 0.850	2.145 ± 1.626	$p < 0.791$
MAOs + COMT	HVA/DA	0.039 ± 0.010	0.046 ± 0.009	$p < 0.141$	0.309 ± 0.094	0.314 ± 0.094	$p < 0.892$	1.821 ± 0.365	2.140 ± 0.577	$p < 0.181$	2.849 ± 1.324	2.763 ± 1.020	$p < 0.872$	1.453 ± 0.413	1.469 ± 0.320	$p < 0.922$

Mean ± SD. Asterisks indicate significance levels: *, $p < 0.05$; **, $p < 0.01$; ***, $p < 0.001$. 3-HAA, 3-hydroxyanthranilic acid; 3-HAO, 3-hydroxyanthranilate oxidase; 3-HK, 3-hydroxykynurenine; AA, anthranilic acid; AADC, aromatic L-amino acid decarboxylase; ACMSD, aminocarboxymuconate-semialdehyde decarboxylase; ALDH, aldehyde dehydrogenase; COMT, catechol-O-methyltransferase; DA, dopamine; DOPAC, 3,4-dihydroxyphenylacetic acid; HVA, homovanillic acid; IAA, indole-3-acetic acid; IDOs, indoleamine 2,3-dioxygenases; INS, indoxyl sulfate; *kat2*$^{-/-}$, kynurenine aminotransferase II knockout mice; KATs, kynurenine aminotransferases; KMO, kynurenine 3-monooxygenase; KYNA, kynurenic acid; KYNU, kynureninase; L-DOPA, dihydroxyphenylalanine/levodopa; MAOs, monoamine oxidases; PA, picolinic acid; QA, quinolinic acid; QAA, quinolinic acid analog; TDO, tryptophan 2,3-dioxygenase; TH, tyrosine hydroxylase; TMO, tryptophan monooxygenase; TNA, tryptophan N-acetyltransferase; TrD, tryptophan deaminase; Trp, tryptophan; Tyr, tyrosine; 5-HIAA, 5-hydroxyindoleacetic acid; 5-HT, serotonin (5-hydroxytryptamine); 5-HTP, 5-hydroxytryptophan; WT, wild-type mice; XA, xanthurenic acid.

Table S6. Ratios of oxidant/antioxidant and N-methyl-D-aspartate (NMDA) agonist/antagonist metabolites across brain regions in wild-type (WT) and *kat2*$^{-/-}$ mice. Regional indices of oxidative stress and excitotoxicity in WT and *kat2*$^{-/-}$ mice.

**Oxidant/antioxidant metabolites**	**Striatum**			**Cortex**			**Hippocampus**			**Cerebellum**			**Brainstem**		
	**Mean ± SD**		***p*-Value**	**Mean ± SD**		***p*-Value**	**Mean ± SD**		***p*-Value**	**Mean ± SD**		***p*-Value**	**Mean ± SD**		***p*-Value**
	**WT**	***kat2*$^{-/-}$**		**WT**	***kat2*$^{-/-}$**		**WT**	***kat2*$^{-/-}$**		**WT**	***kat2*$^{-/-}$**		**WT**	***kat2*$^{-/-}$**	
3-HK/(KYNA+AA+XA)	6.951 ± 2.904	10.723 ± 10.215	$p < 0.627$	4.700 ± 2.112	17.670 ± 13.315	$p < 0.001$ **	13.509 ± 6.992	37.148 ± 15.859	$p < 0.002$ **	9.667 ± 3.839	20.311 ± 9.275	$p < 0.006$ **	5.740 ± 1.892	18.705 ± 9.300	$p < 0.002$ **
**NMDA agonist/antagonist metabolites**	**Striatum**			**Cortex**			**Hippocampus**			**Cerebellum**			**Brainstem**		
	**Mean ± SD**		***p*-value**	**Mean ± SD**		***p*-value**	**Mean ± SD**		***p*-value**	**Mean ± SD**		***p*-value**	**Mean ± SD**		***p*-value**
	**WT**	***kat2*$^{-/-}$**		**WT**	***kat2*$^{-/-}$**		**WT**	***kat2*$^{-/-}$**		**WT**	***kat2*$^{-/-}$**		**WT**	***kat2*$^{-/-}$**	
QA/KYNA	11.575 ± 18.379	9.138 ± 7.260	$p < 0.923$	9.263 ± 7.574	14.235 ± 14.254	$p < 0.597$	7.957 ± 5.478	16.120 ± 9.907	$p < 0.046$ *	5.306 ± 4.601	4.998 ± 4.789	$p < 0.880$	7.724 ± 7.414	10.224 ± 5.544	$p < 0.096$

Mean ± SD. Asterisks indicate significance levels: *, $p < 0.05$; **, $p < 0.01$; ***, $p < 0.001$. 3-HK, 3-hydroxykynurenine; AA, anthranilic acid; *kat2*$^{-/-}$, kynurenine aminotransferase II knockout mice; KYNA, kynurenic acid; NMDA. N-methyl-D-aspartate; QA, quinolinic acid; WT, wild-type mice; XA, xanthurenic acid.
